# TMT-based quantitative proteomics analysis reveals the attenuated replication mechanism of Newcastle disease virus caused by nuclear localization signal mutation in viral matrix protein

**DOI:** 10.1080/21505594.2020.1770482

**Published:** 2020-06-27

**Authors:** Zhiqiang Duan, Chao Yuan, Yifan Han, Lei Zhou, Jiafu Zhao, Yong Ruan, Jiaqi Chen, Mengmeng Ni, Xinqin Ji

**Affiliations:** a Key Laboratory of Animal Genetics, Breeding and Reproduction in the Plateau Mountainous Region, Ministry of Education, Guizhou University, Guiyang, China; b College of Animal Science, Guizhou University, Guiyang, China

**Keywords:** Newcastle disease virus, matrix protein, nuclear localization signal, nucleocytoplasmic trafficking, TMT, quantitative proteomics

## Abstract

Nuclear localization of cytoplasmic RNA virus proteins mediated by intrinsic nuclear localization signal (NLS) plays essential roles in successful virus replication. We previously reported that NLS mutation in the matrix (M) protein obviously attenuates the replication and pathogenicity of Newcastle disease virus (NDV), but the attenuated replication mechanism remains unclear. In this study, we showed that M/NLS mutation not only disrupted M’s nucleocytoplasmic trafficking characteristic but also impaired viral RNA synthesis and transcription. Using TMT-based quantitative proteomics analysis of BSR-T7/5 cells infected with the parental NDV rSS1GFP and the mutant NDV rSS1GFP-M/NLSm harboring M/NLS mutation, we found that rSS1GFP infection stimulated much greater quantities and more expression changes of differentially expressed proteins involved in host cell transcription, ribosomal structure, posttranslational modification, and intracellular trafficking than rSS1GFP-M/NLSm infection. Further in-depth analysis revealed that the dominant nuclear accumulation of M protein inhibited host cell transcription, RNA processing and modification, protein synthesis, posttranscriptional modification and transport; and this kind of inhibition could be weakened when most of M protein was confined outside the nucleus. More importantly, we found that the function of M protein in the cytoplasm effected the inhibition of TIFA expression in a dose-dependent manner, and promoted NDV replication by down-regulating TIFA/TRAF6/NF-κB-mediated production of cytokines. It was the first report about the involvement of M protein in NDV immune evasion. Taken together, our findings demonstrate that NDV replication is closely related to the nucleocytoplasmic trafficking of M protein, which accelerates our understanding of the molecular functions of NDV M protein.

## Introduction

*Paramyxoviridae* family includes several enveloped viruses with non-segmented negative-sense RNA genomes that can cause serious diseases in humans and animals, such as Sendai virus (SeV), measles virus (MeV), mumps virus (MuV), Nipah virus (NiV), Hendra virus (HeV), parainfluenza virus types 1–5 (PIV 1–5), and Newcastle disease virus (NDV) [1,2]. As an important member in the genus *Avulavirus* of the family *Paramyxoviridae*, NDV is a highly infectious agent of avian species and may cause devastating losses in the poultry industry worldwide. The genome of NDV is approximately 15.2 kb in length and encodes eight proteins, including six structural proteins (nucleocapsid protein (NP), phosphoprotein protein (P), matrix protein (M), fusion protein (F), hemagglutinin-neuraminidase protein (HN) and large polymerase protein (L)) as well as two non-structural proteins (V and W) derived from the RNA editing of the P gene [1]. Like most of the *Paramyxoviridae* family members, although NDV completes its life cycle in the cytoplasm, both M protein and W protein of NDV can localize to the nucleus via a bipartite nuclear localization signal (NLS) at specific times in virus-infected cells [3,4]. However, unlike the nuclear localization and unknown function of W protein, the NDV M protein is demonstrated to be a nucleocytoplasmic shuttling protein and plays crucial roles in NDV life cycle [1]. In addition to participating in the assembly in the cytoplasm and budding of progeny virions at the cell membrane later in infection [5], the NDV M protein is localized in the nucleus and nucleolus early in infection [6], which is thought to inhibit host cell transcription and protein synthesis similar to the M protein of human respiratory syncytial virus (HRSV), vesicular stomatitis virus (VSV) and MeV [7–9]. Recent studies have shown that the nuclear import of NDV M protein is mediated by its NLS region (^247^KKGKKVIFDKIEEKIRR^263^) interacting with importin β1 via the RanGTP-dependent pathway [10], and the nuclear export of NDV M protein is mediated by three nuclear export signals (NESs) via the CRM1-independent pathway [11]. But so far, there is limited information about the biological functions of M’s nucleocytoplasmic trafficking of NDV and other *Paramyxoviridae* family members.

Increasing lines of evidence have suggested that the nuclear localization and nuclear export of cytoplasmic RNA virus proteins plays essential roles in successful virus replication by inhibiting antiviral response or promoting virus budding [12–15]. For example, nuclear localization of porcine respiratory and reproductive syndrome virus (PRRSV) nucleocapsid protein is found to benefit for optimal virus replication and inhibiting cellular antiviral processes [16], and nuclear export of PRRSV nonstructural protein 1α is necessary for type I IFN inhibition [17]. Meanwhile, nuclear localization of HRSV M protein is important for inhibiting cell transcription and is associated with the pathogenesis of virus infection [7,12], and nuclear export-deficient of HRSV M protein fails to localize to regions of virus assembly and thereby absolutely inhibits virus replication [18]. In addition, the interaction of phospholipid scramblase 1 with influenza A virus (IAV) NP protein inhibits the incorporation of importin β into the importin α/β complex, which reduces the nuclear import of NP and suppresses virus replication [19], and inhibition of CRM1-mediated nuclear export of IAV NP protein and nuclear export protein impairs virus budding [20]. Moreover, a previous study has also revealed that ubiquitin-regulated nucleocytoplasmic trafficking of the NiV M protein is associated with M’s post-translational modification and plays a critical role in M-mediated viral budding [21]. In our recent studies, we demonstrated that a recombinant NDV with NLS mutation (^247^AAGAAVIFDKIEEKIAA^263^) in the M protein not only resulted in a pathotype change of virulent NDV but also significantly attenuated the replication and pathogenicity of NDV in chicken fibroblasts and SPF chickens [10]. More importantly, a recombinant NDV carrying mutated M/NESs can not be rescued by reverse genetics due to the core role of M protein in viral assembly and budding in the cytoplasm [11]. These results clearly indicated that nucleocytoplasmic trafficking of M protein was closely related to the replication and pathogenicity of NDV.

Proteomics analysis of host cells responding to virus infection are important tools in identifying cellular proteins involved directly or indirectly in virus replication and helping to understand how virus infection leads to host pathogenicity [22,23]. In recent years, tandem mass tag (TMT) combined with liquid chromatography tandem-mass spectrometry (LC-MS/MS) analysis has become a powerful tool in the identification, characterization, and quantitation analysis of the proteomic profiles [24,25]. This approach has been successfully applied to many virus-host interaction studies, such as in human immunodeficiency virus (HIV) [26,27], Bombyx mori nucleopolyhedrovirus [28], canine parvovirus [29], HIV/TM co-infected patients [30], and Epstein-Barr virus [31]. These studies have revealed the dynamic interactions between virus and host, and provided a better understanding of the pathogenesis involved in viral infection and replication. Up to now, studies about proteomic changes in host cells or tissues infected with NDV mainly focus on the pathogenesis of NDV-infected chicken peripheral blood mononuclear cells [32], NDV/infectious bronchitis coronavirus/H9 subtype avian influenza virus co-infected chicken tracheal [33], NDV-infected chicken lung during heat stress [34], and NDV-caused extracellular matrix degradation and immunopathology in chicken spleen [35]. However, there have been few comparative proteomics studies investigating the host interaction with the wild type NDV and its attenuated NDV caused by amino acids mutation in viral proteins. Therefore, in this study, we employed a quantitative proteomics analysis based on TMT coupled with LC-MS/MS to screen the differential protein expression profiles of BSR-T7/5 cells upon infection with the parental NDV (rSS1GFP) and the mutant NDV (rSS1GFP-M/NLSm) carrying M/NLS mutation. The results will provide valuable information for better understanding the attenuated replication mechanism of NDV caused by NLS mutation in M protein and the potential biological functions of NDV M’s nucleocytoplasmic trafficking, and also accelerate our understanding of the molecular mechanisms underlying the replication and pathogenesis of NDV.

## Results

### Nucleocytoplasmic trafficking of M protein promotes the replication and cytopathogenicity of NDV and regulates viral RNA synthesis and transcription

We previously reported that M/NLS mutation disrupted the nuclear localization of M protein and attenuated the replication efficiency and plaque formation ability of NDV in chicken fibroblasts [10]. To learn about the effect of M/NLS mutation on the dynamic changes of M’s intracellular localization in more detail, we compared the subcellular localization of M protein in rSS1GFP- and rSS1GFP-M/NLSm-infected BSR-T7/5 cells at different time points. As shown in Figure 1(a), at early time points, the M protein of rSS1GFP was primarily concentrated in the nucleolus with a discrete punctuate staining pattern at 6 hour post-infection (hpi), and then was observed in the largest concentration in the nucleus and nucleolus at 12 hpi. While at later time points, the rSS1GFP M protein was distributed diffusely in the cytoplasm, with some still localized in the nucleolus at 18 and 24 hpi. By contrast, most of the rSS1GFP-M/NLSm M protein accumulated around the nucleus at 6 and 12 hpi, and then localized exclusively in the cytoplasm at 18 and 24 hpi (Figure 1(a)). Interestingly, the appearance of large inclusion bodies induced by the membrane fusion of rSS1GFP-infected cells was clearly observed through the fluorescence of GFP and DAPI at 24 hpi (Figure 1(a)). The replication ability and cytopathogenicity of rSS1GFP and rSS1GFP-M/NLSm in BSR-T7/5 cells were then evaluated. The results of multicycle growth kinetics showed that the virus titers of rSS1GFP-M/NLSm were remarkably lower than that of rSS1GFP from 12 to 48 hpi (*P* < 0.001) (Figure 1(b)). In addition, the cytopathic effect (CPE) in rSS1GFP-infected cells started at 12 hpi and cell monolayer began to be destroyed at 24 hpi, but the slight CPE in rSS1GFP-M/NLSm-infected cells started to appear and cell monolayer destruction was not examined at 24 hpi (Figure 1(c)). Moreover, the GFP fluorescence in rSS1GFP-infected cells was also much brighter and more than that in rSS1GFP-M/NLSm-infected cells at the same time points (Figure 1(c)). These data suggested that M/NLS mutation disrupted the nucleocytoplasmic trafficking of M protein and weakened the replication and cytopathogenicity of NDV.

**Figure 1.**
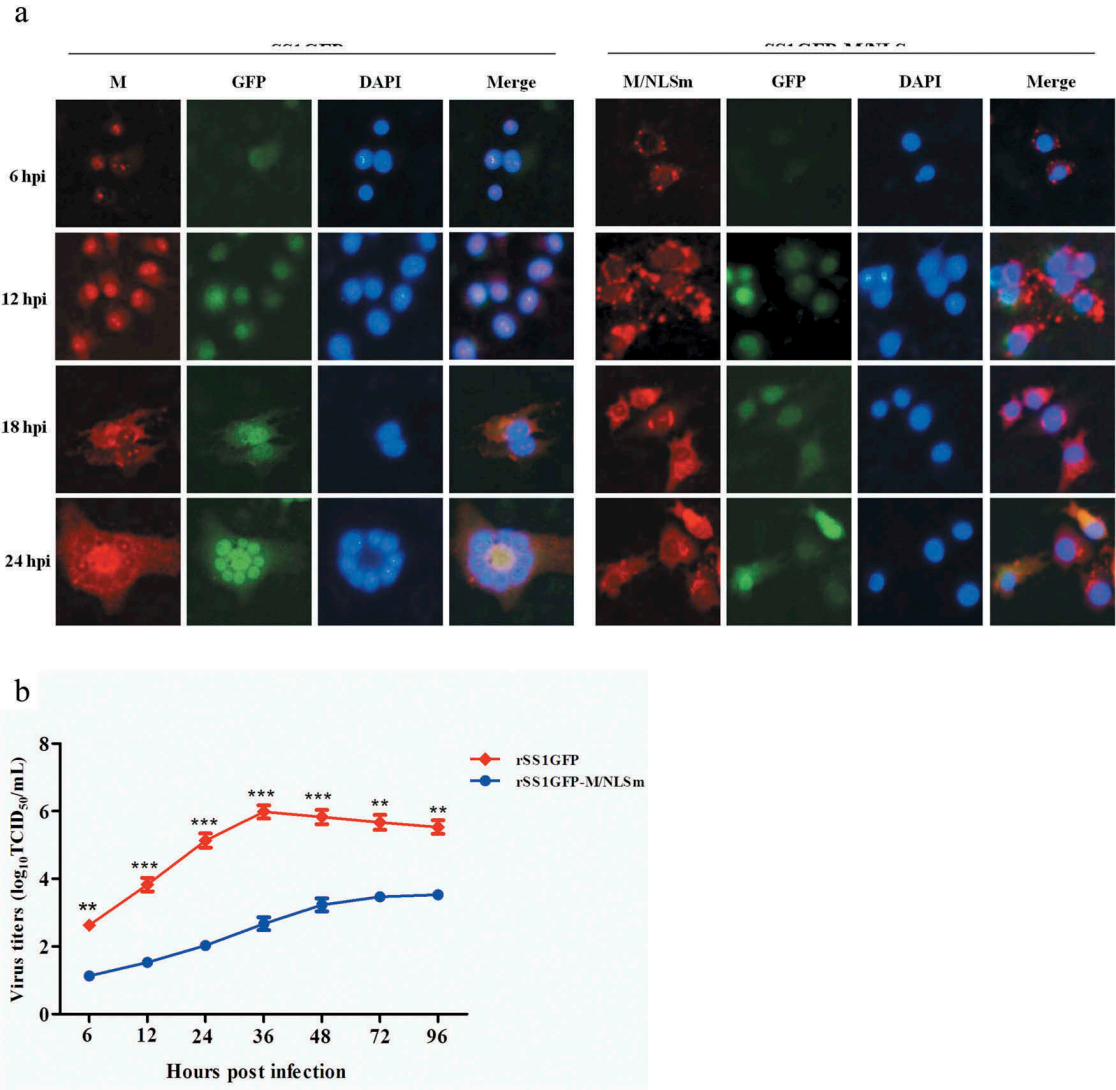
Nucleocytoplasmic trafficking of M protein promotes the replication and cytopathogenicity of NDV by affecting viral RNA synthesis and transcription. (a) The subcellular localization of M protein in rSS1GFP- and rSS1GFP-M/NLSm-infected BSR-T7/5 cells at 6, 12, 18 and 24 hpi. DAPI was used to stain nuclei. Original magnification was 1 × 200. (b) Virus titers were detected in BSR-T7/5 cells at the indicated time points. (c) The CPE and GFP were observed in virus-infected BSR-T7/5 cells at 12 and 24 hpi. Original magnification was 1 × 200. (d) The viral RNA synthesis corresponding to the NP and P genes and (e) viral transcription corresponding to the M and GFP genes in rSS1GFP- and rSS1GFP-M/NLSm-infected BSR-T7/5 cells were detected by qRT-PCR. (f) The expression levels of NP, M and GFP proteins in rSS1GFP- and rSS1GFP-M/NLSm-infected BSR-T7/5 cells were examined by Western blotting. The relative levels of the NP, M and GFP proteins were compared with the control GAPDH expression. Each data indicates the mean ± SD of three independent experiments. *P*-values are indicated by asterisks (*P < 0.05, **P < 0.01, ***P < 0.001 compared to the value of rSS1GFP-M/NLSm).

**Figure 1.**
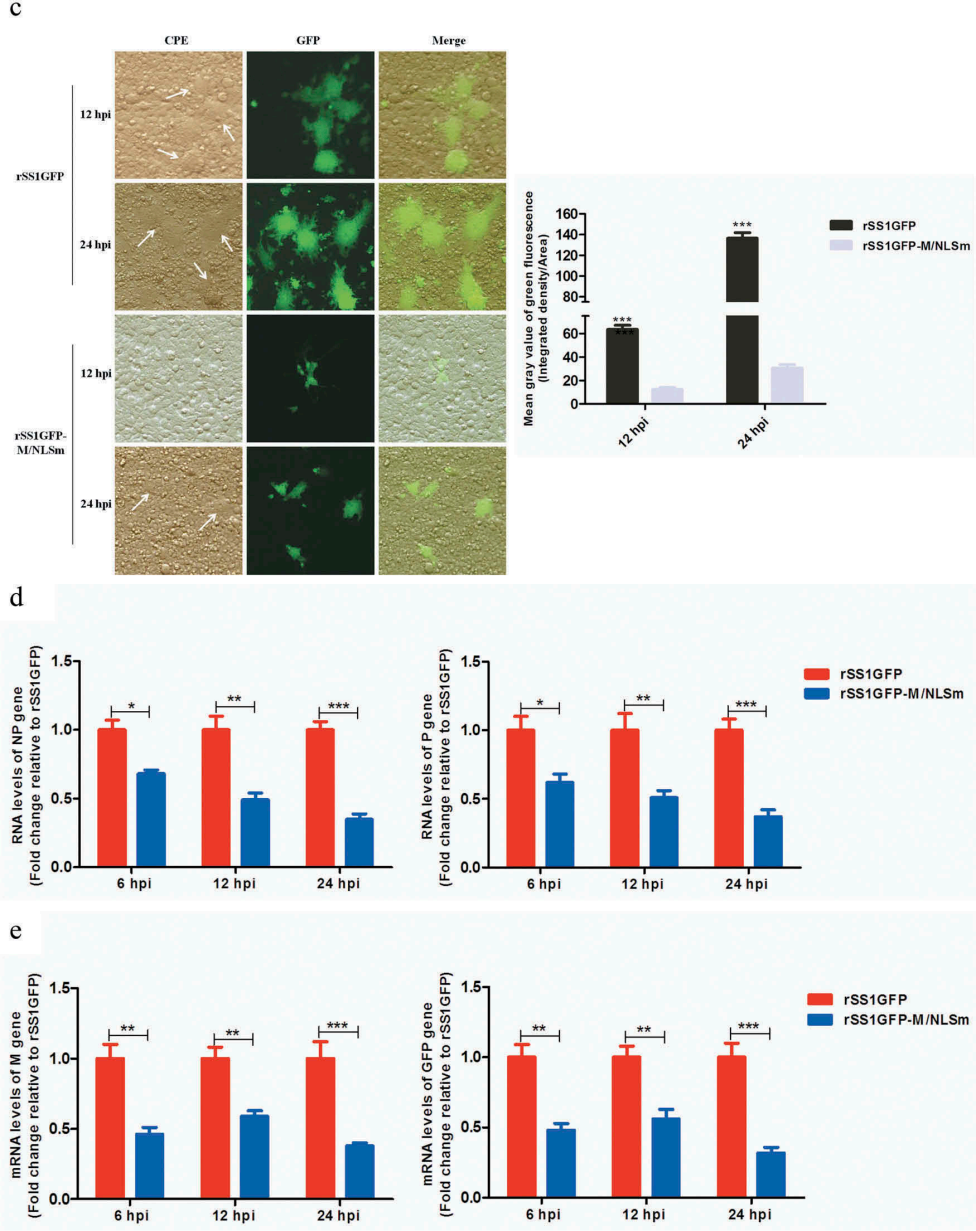
(Continued).

**Figure 1.**
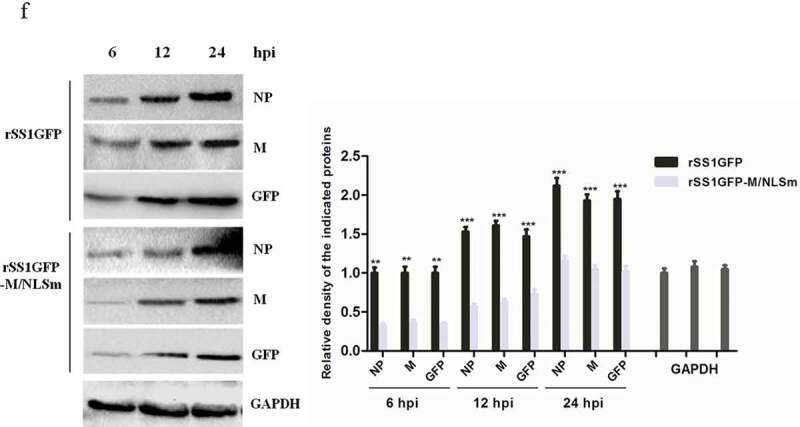
(Continued).

To investigate whether the attenuated replication and cytopathogenicity of rSS1GFP-M/NLSm due to the reduced viral RNA synthesis and transcription, the RNA levels of NP and P genes and the mRNA levels of M and GFP genes in rSS1GFP- and rSS1GFP-M/NLSm-infected cells were analyzed by quantitative real-time PCR (qRT-PCR). The results showed that the relative RNA levels (corresponding to the NP and P genes) between rSS1GFP- and rSS1GFP-M/NLSm-infected cells had statistically significant differences at 6 hpi (*P* < 0.05), which continued to reduce in rSS1GFP-M/NLSm-infected cells at 12 and 24 hpi (Figure 1(d)). Similarly, the relative mRNA levels of M and GFP genes in rSS1GFP-M/NLSm-infected cells were more decreased than that in rSS1GFP-infected cells at 6 and 12 hpi (*P* < 0.01), and significantly lower at 24 hpi (*P* < 0.001) (Figure 1(e)). Consistent with the relative mRNA expression levels of M and GFP genes, the expression levels of NP, M and GFP proteins were also greatly reduced during the course of rSS1GFP-M/NLSm infection when compared to rSS1GFP infection (figure 1(f)). Together, these results indicated that nucleocytoplasmic trafficking of M protein promoted the replication and cytopathogenicity of NDV possibly by affecting viral RNA synthesis and transcription.

### rSS1GFP and rSS1GFP-M/NLSm exhibit great discrepancy in stimulating the cellular proteome

**Figure 2.**
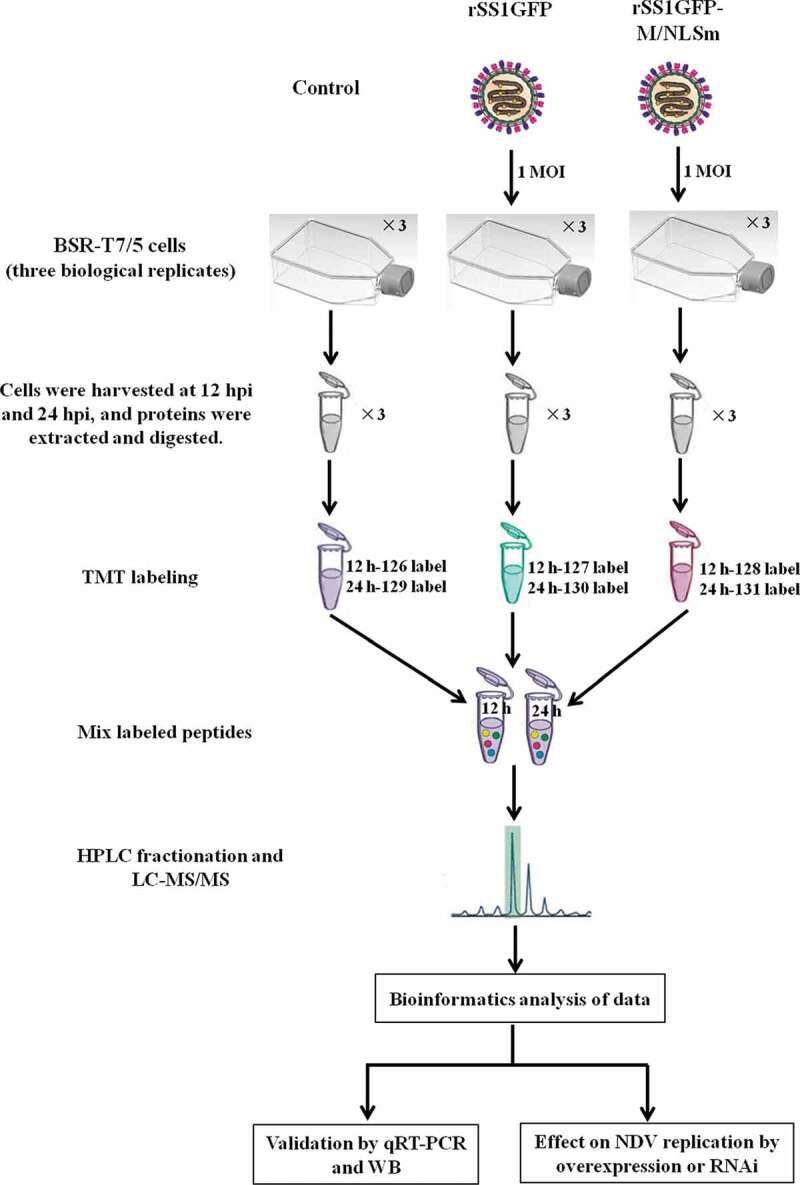
Workflow for TMT-coupled LC-MS/MS analysis of BSR-T7/5 cells infected with rSS1GFP and rSS1GFP-M/NLSm viruses.

**Figure 3.**
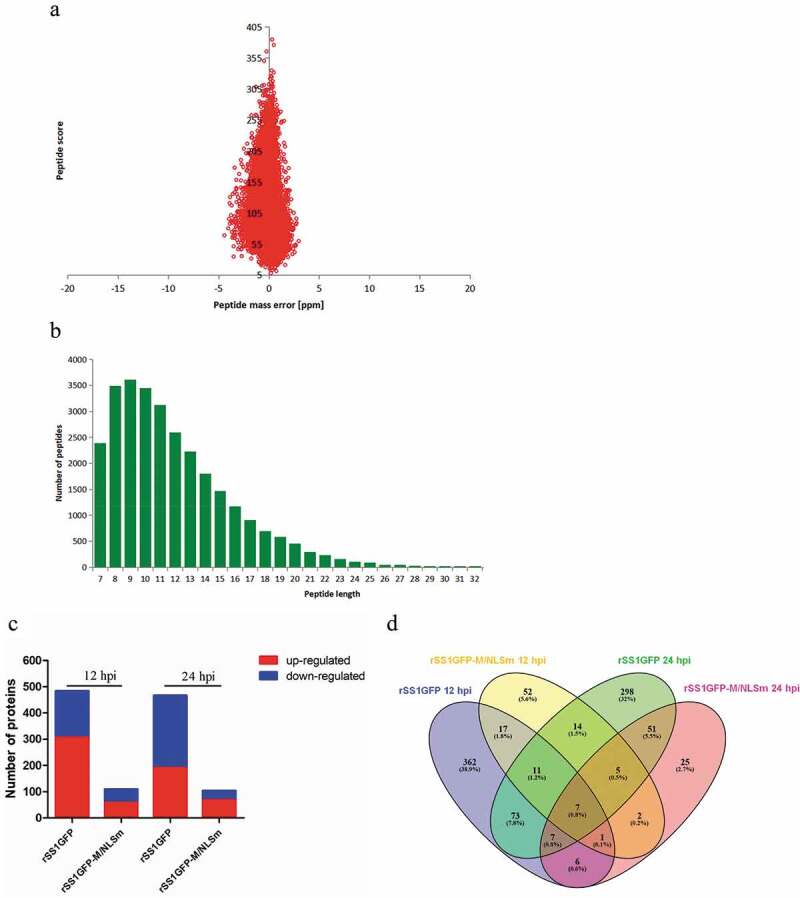
MS identified information based on proteomics analysis of DEPs in BSR-T7/5 cells infected with rSS1GFP and rSS1GFP-M/NLSm. (A) Average peptide mass error. (B) All identified peptides length distribution. (C) Numbers of DEPs during infection with rSS1GFP or rSS1GFP-M/NLSm virus relative to mock infection at 12 and 24 hpi. (D) The distribution of DEPs during rSS1GFP or rSS1GFP-M/NLSm infection at 12 and 24 hpi using Venn diagram analysis.

To further investigate the mechanism underlying the attenuated replication of rSS1GFP-M/NLSm, the global cellular protein expression profiles of BSR-T7/5 cells infected with these two viruses at 12 and 24 hpi was compared (Figure (2)). After quality validation, a total of 530,600 (111,751 matched, accounted for 21.1%) spectra were obtained, of which 59,112 were identified peptides (56,897 unique peptides) and 5899 were identified proteins (5308 quantified proteins) (Table 1). The average peptides mass error was less than 10 ppm (Figure 3(a)), suggesting a high mass accuracy of the MS data. The length of most identified peptides mainly distributed from 7 to 20 amino acid residues (Figure 3(b)), which indicated that these samples met the required standard. The detailed information of identified proteins, including protein accession, protein description, gene name, coverage, peptide number, peptide-spectrum match, carried charges and so on, is shown in Supplemental material Table S1. In addition, significantly up- or down-regulated proteins were determined by fold-change ratios >1.2 or <0.83 and *P* < 0.05. The results showed that 484 and 466 proteins from the rSS1GFP group displayed significantly altered expression levels compared with the normal control at 12 and 24 hpi, respectively, including 306 up-regulated proteins and 178 down-regulated proteins at 12 hpi, and 190 up-regulated proteins and 276 down-regulated proteins at 24 hpi (Figure 3(c), Supplemental material Table S2). But as for the rSS1GFP-M/NLSm group, only 109 and 104 proteins were significantly differentially expressed at 12 and 24 hpi, respectively, including 58 up-regulated proteins and 51 down-regulated proteins at 12 hpi, and 68 up-regulated proteins and 36 down-regulated proteins at 24 hpi (Figure 3(c), Supplemental material Table S2). A Venn diagram summarizing the distribution of differentially expressed proteins (DEPs) revealed that 36 and 70 DEPs were shared by rSS1GFP and rSS1GFP-M/NLSm at 12 and 24 hpi, respectively (Figure 3(d), Supplemental material Table S3 and S4). However, only seven DEPs were jointly shared by these two viruses at 12 and 24 hpi (Figure 3(d) and Table 2). In addition, top 10 up-regulated and down-regulated significantly DEPs induced by rSS1GFP and rSS1GFP-M/NLSm at 12 hpi and 24 hpi were listed in Supplemental material Table S5 and Table S6, respectively, showing that rSS1GFP caused much higher ratio of up-regulated DEPs or much lower ratio of down-regulated DEPs than rSS1GFP-M/NLSm. These results suggested that rSS1GFP infection stimulated much greater quantities and more expression changes of DEPs than rSS1GFP-M/NLSm infection.

**Table 1.** Summary of MS/MS spectrum database search analysis.

Total spectrum	Matched spectrum	Peptides	Unique peptides	Identified proteins	Quantifiable proteins
530,600	111,751 (21.1%)	59,112	56,897	5899	5308

**Table 2.** Significantly DEPs jointly shared by rSS1GFP and rSS1GFP-M/NLSm at 12 hpi and 24 hpi.

			12 hpi				24 hpi			
			rSS1GFP		rSS1GFP-M/NLSm		rSS1GFP		rSS1GFP-M/NLSm	
Protein accession	Protein description	Gene symbol	Ratio^a^	*p*-value^b^	Ratio^a^	*p*-value^b^	Ratio^a^	*p*-value^b^	Ratio^a^	*p*-value^b^
G3IJM1	TRAF-interacting protein with FHA domain-containing protein A	TIFA	1.803	0.030791	1.322	0.046455	0.271	0.0295203	1.267	0.031014
G3IL75	Collagen alpha-1(V) chain	COL5A1	1.273	0.014047	1.260	0.021040	1.226	0.0097879	1.254	0.032310
G3HW17	Cell division cycle 5-like protein	CDC5 L	1.243	0.000402	1.239	0.040423	0.830	0.0204819	0.805	0.046613
G3HX39	Phosphoglycerate kinase	PGKB	0.776	0.047680	0.815	0.009816	0.705	0.0170213	0.760	0.007895
G3IN18	60S ribosomal protein L8	RPL8	0.746	0.038874	0.815	0.004297	1.642	0.0179714	1.242	0.021739
G3H5N3	60S ribosomal protein L29	RPL29	0.667	0.038259	0.817	0.040208	1.847	0.0305984	1.418	0.011640
G3ILI7	26S proteasome non-ATPase regulatory subunit 3	PSMD3	0.529	0.017975	0.652	0.047559	0.790	0.0284991	1.329	0.002257

^a^means average value of rSS1GFP- or rSS1GFP-M/MNLSm-infected group relative to the control group.

^b^means one-sample *t* test *p-*value.

### Annotation analysis of the identified DEPs

**Figure 4.**
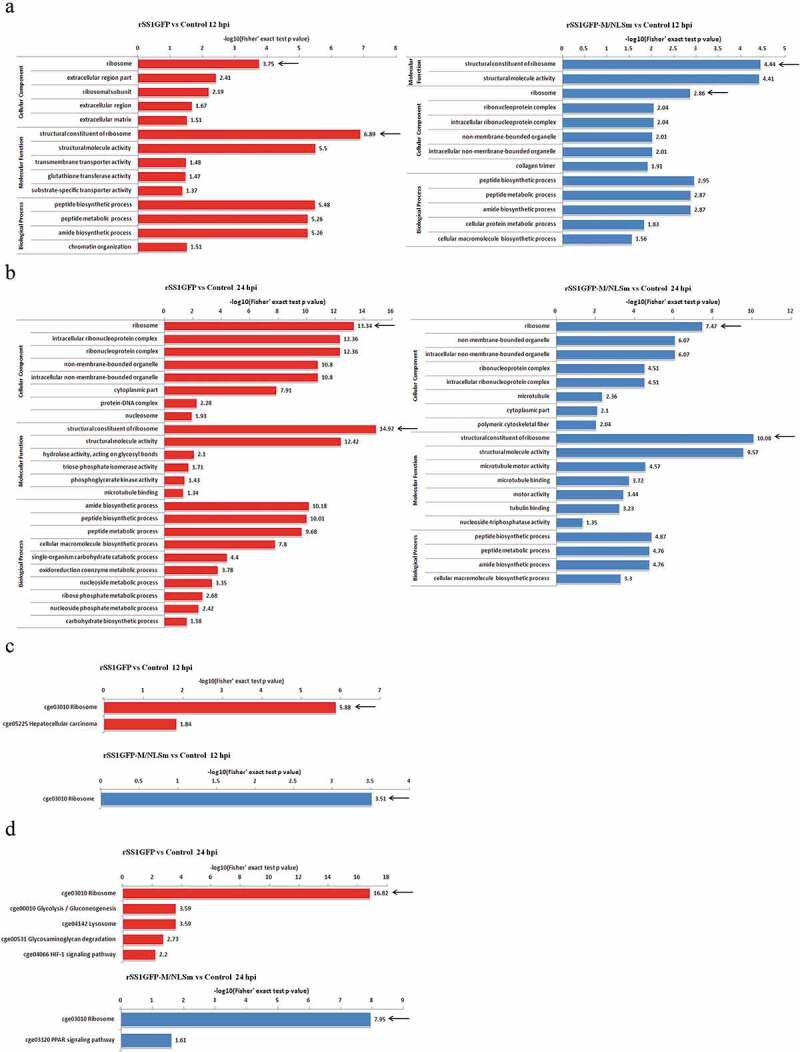
Enrichment analysis of the identified DEPs during rSS1GFP and rSS1GFP-M/NLSm infection . GO enrichment analysis on the ontology of cellular component, biological process and molecular function during rSS1GFP and rSS1GFP-M/NLSm infection at 12 hpi (a) and 24 hpi (b), respectively. KEGG enrichment analysis of the DEPs during rSS1GFP and rSS1GFP-M/NLSm infection at 12 hpi (c) and 24 hpi (d), respectively.

DEPs were then annotated to several Gene Ontology (GO) analysis including cellular component, biological process and molecular function. GO enrichment analysis showed that the most significantly enriched cellular components for rSS1GFP group were the ribosome, extracellular region part and ribosomal subunit at 12 hpi, and the ribosome, intracellular ribonucleoprotein complex and ribonucleoprotein complex at 24 hpi; but the main enriched cellular components for rSS1GFP-M/NLSm group were the ribosome, ribonucleoprotein complex and intracellular ribonucleoprotein complex at 12 hpi, and the ribosome, non-membrane-bounded organelle and intracellular non-membrane-bounded organelle at 24 hpi (Figure 4(a,b)). As for the biological process enrichment, the peptide biosynthesis process, peptide metabolic process and amide biosynthesis process were commonly found in both rSS1GFP group and rSS1GFP-M/NLSm group at 12 and 24 hpi (Figure 4(a,b)). Meanwhile, the structural constitute of ribosome and structural molecule activity were the enriched molecular functions commonly found in the two groups at 12 and 24 hpi (Figure 4(a,b)). To obtain more information about the biological pathways in which the DEPs may be involved, KEGG pathway enrichment analysis was also compared. As shown in Figure 4(c,d), the ribosome pathway was the co-owned signaling pathway existed in rSS1GFP group and rSS1GFP-M/NLSm group at 12 and 24 hpi. But beyond that, the DEPs in rSS1GFP group were also largely enriched in the glycolysis/gluconeogenesis, lysosome, glycosaminoglycan degradation and HIF-1 signaling pathways 24 hpi (Figure 4(d)).

**Figure 5.**
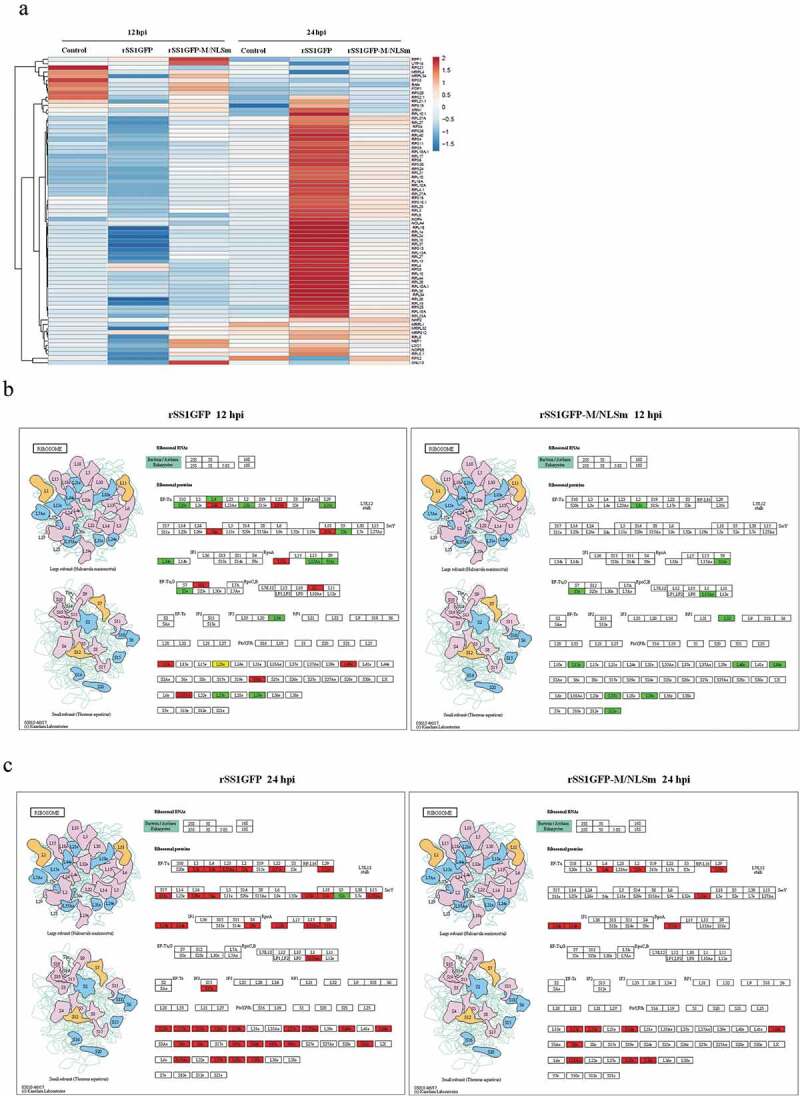
Analysis of the ribosome-related protein changes during rSS1GFP and rSS1GFP-M/NLSm infection. (a) The hierarchical clustering heatmap of ribosome-related proteins during rSS1GFP and rSS1GFP-M/NLSm infection at 12 and 24 hpi. The color scale bar locates in the right, and blue and red indicate decreased and increased levels of the identified DEPs, respectively. The modeling of ribosome signaling pathway in BSR-T7/5 cells infected with rSS1GFP (b) or rSS1GFP-M/NLSm (c) at 12 and 24 hpi, respectively (red, up-regulated DEPs; green, down-regulated DEPs; yellow, both up-regulated and down-regulated DEPs).

It is remarkable that the DEPs were jointly enriched in the ribosome (Cellular Component), structural constitute of ribosome (Molecular Function) and ribosome pathway (KEGG pathway) in rSS1GFP group and rSS1GFP-M/NLSm group at 12 and 24 hpi (Figure 4, indicated by arrow). Therefore, the dynamic changes of ribosome-related DEPs in BSR-T7/5 cells infected with rSS1GFP and rSS1GFP-M/NLSm at 12 and 24 hpi were compared. As shown in Figure 5(a), the hierarchical clustering heatmap of ribosome-related DEPs in rSS1GFP group displayed more changes (most of DEPs were significantly down-regulated at 12 hpi and up-regulated at 24 hpi) than rSS1GFP-M/NLSm group. In addition, the modeling of ribosome pathway showed that almost equivalent amount of up-regulated and down-regulated DEPs were present in rSS1GFP group at 12 hpi, while a small number of down-regulated DEPs appeared in rSS1GFP-M/NLSm group at 12 hpi (Figure 5(b)). Although the DEPs in both rSS1GFP and rSS1GFP-M/NLSm group exhibited up-regulated, the number of DEPs in rSS1GFP group were much more than that in rSS1GFP-M/NLSm group (Figure 5(c)).

**Figure 6.**
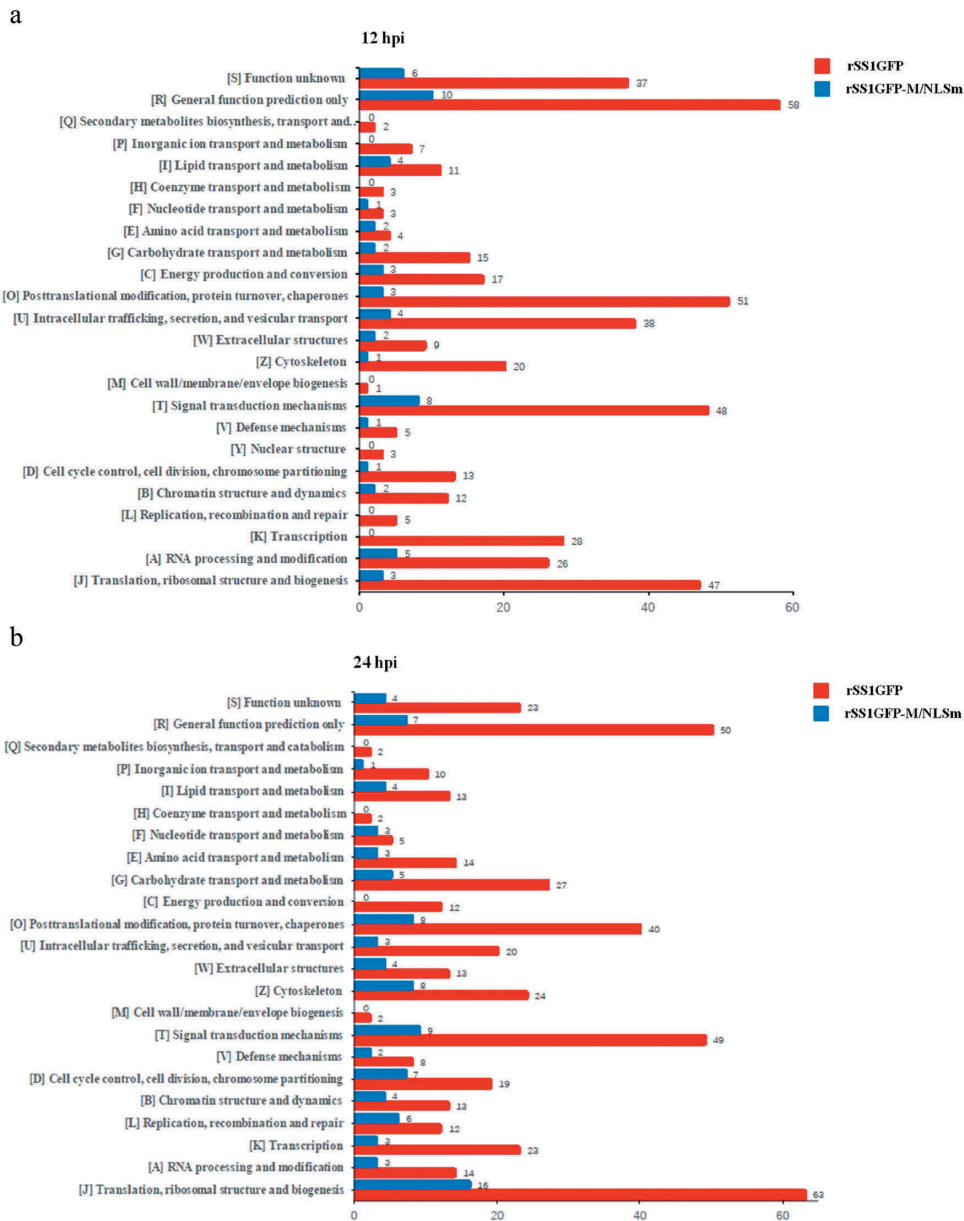
COG/KOG functional classification distribution maps of DEPS in BSR-T7/5 cells infected with rSS1GFP and rSS1GFP-M/NLSm at 12 hpi (a) and 24 hpi (b), respectively. A total of 24 groups of DEPs were clustered based on orthologous groups.

Clusters of orthologous groups (COG)/clusters of eukaryotic orthologous groups (KOG) categories of the identified DEPs in rSS1GFP and rSS1GFP-M/NLSm groups was further analyzed. The results showed that “General function prediction only” (group R, 58 DEPs), “Posttranslational modification, protein turnover, chaperones” (group O, 51 DEPs), and “Translation, ribosomal structure and biogenesis” (group J, 63 DEPs), “General function prediction only” (group R, 50 DEPs) represented the four largest groups in rSS1GFP group at 12 and 24 hpi, respectively (Figure 6(a,b)). However, “Translation, ribosomal structure and biogenesis” (group J, 15 DEPs), “Signal transduction mechanisms” (group T, 12 DEPs), and “Translation, ribosomal structure and biogenesis” (group J, 16 DEPs), “Signal transduction mechanisms” (group T, 9 DEPs) were the largest four groups in rSS1GFP-M/NLSm group at 12 and 24 hpi, respectively (Figure 6(a,b)). Overall, the annotation analysis of DEPs revealed that rSS1GFP infection could induce more function and signaling pathway changes of DEPs than rSS1GFP-M/NLSm infection.

### rSS1GFP infection inhibits host cell transcription, RNA processing and modification

It has been reported that the M protein of several non-segmented negative-sense RNA viruses (NNSVs) including HRSV, VSV and MeV has the ability to inhibit host cell transcription in various ways [7–9]. To determine whether the NDV M protein can inhibit cell transcription process, we mainly focused on the expression profiles of DEPs related to “Transcription” and “RNA processing and modification” according to the results of COG/KOG categories analysis. A total of 53 and 39 DEPs associated with “Transcription” and “RNA processing and modification” were found in rSS1GFP group, respectively, but 20 representative DEPs of each category were selected for further analysis. The results showed that the expression of most DEPs related to “Transcription” and “RNA processing and modification” was significantly decreased in rSS1GFP group at 12 hpi, and was still slightly decreased at 24 hpi (Figure 7(a,b)). On the contrary, the expression of many DEPs related to two processes showed up-regulated in rSS1GFP-M/NLSm group at 12 hpi, and especially exhibited much higher up-regulation in “Transcription” at 24 hpi (Figure 7(a,c)).

**Figure 7.**
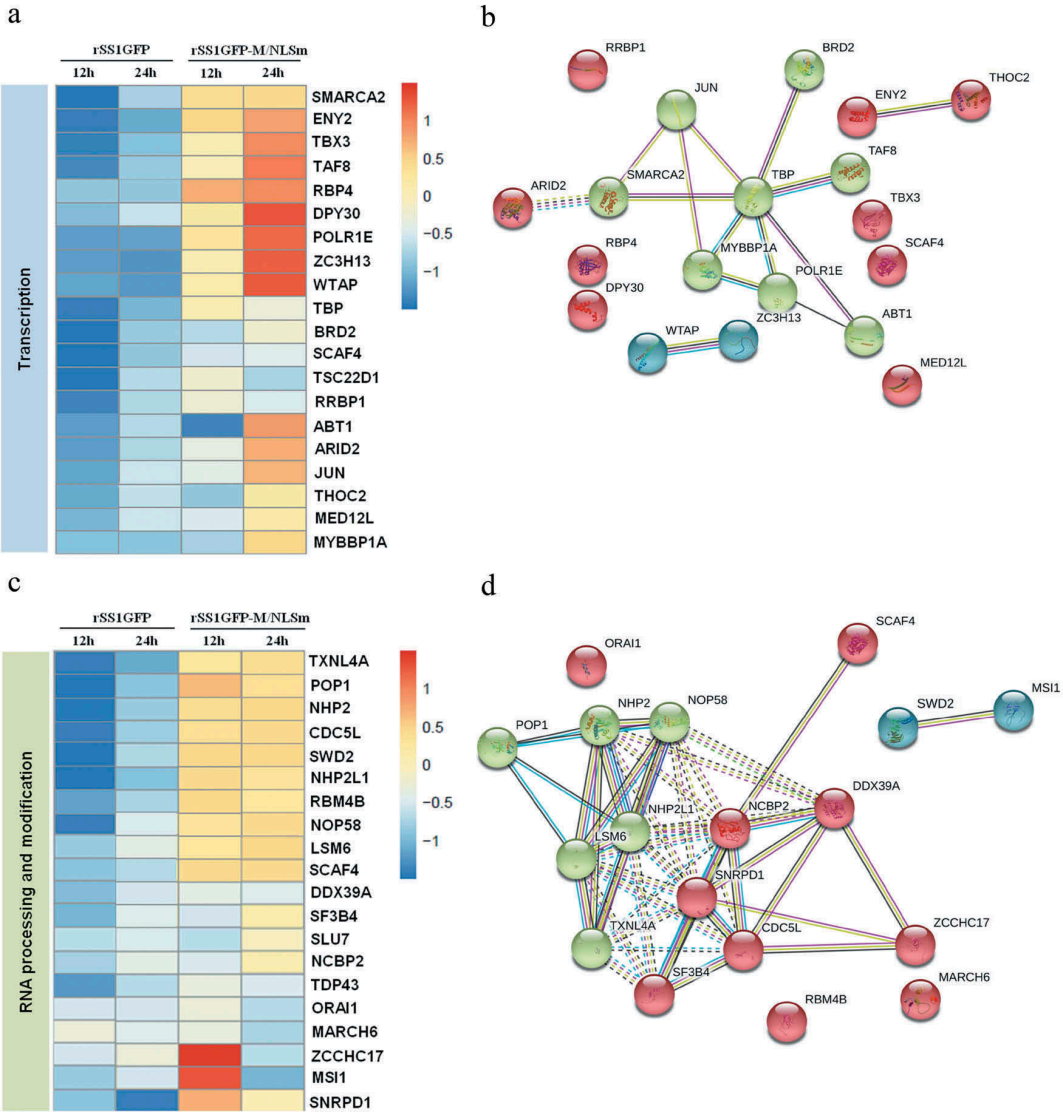
rSS1GFP infection inhibits host cell transcription, RNA processing and modification. (a) The heatmap of representative 20 DEPs related to “Transcription” during rSS1GFP and rSS1GFP-M/NLSm infection at 12 and 24 h. (b) The protein-protein interactions of the DEPs related to “Transcription” are analyzed by the STRING software. A red line indicates the presence of fusion evidence; a blue line indicates co-occurrence evidence; a light blue line indicates database evidence; a purple line indicates experimental evidence; a green line indicates neighborhood evidence; a black line indicates co-expression evidence. (c) The heatmap of representative 20 DEPs related to “RNA processing and modification” during rSS1GFP and rSS1GFP-M/NLSm infection at 12 and 24 h. (d) The protein-protein interactions of the DEPs related to “RNA processing and modification” are analyzed by the STRING software. (e) The mRNA expression levels of four selected DEP genes in BSR-T7/5 cells infected with rSS1GFP and rSS1GFP-M/NLSm were verified by qRT-PCR. (f) The protein expression levels of four DEPs in BSR-T7/5 cells infected with rSS1GFP and rSS1GFP-M/NLSm were examined by Western blotting. The relative expression levels of four DEPs were compared with the control GAPDH expression. Error bars represent standard deviations (mean ± SD) (*P < 0.05; **P < 0.01; ***P < 0.001 compared to the value of rSS1GFP-M/NLSm).

**Figure 7.**
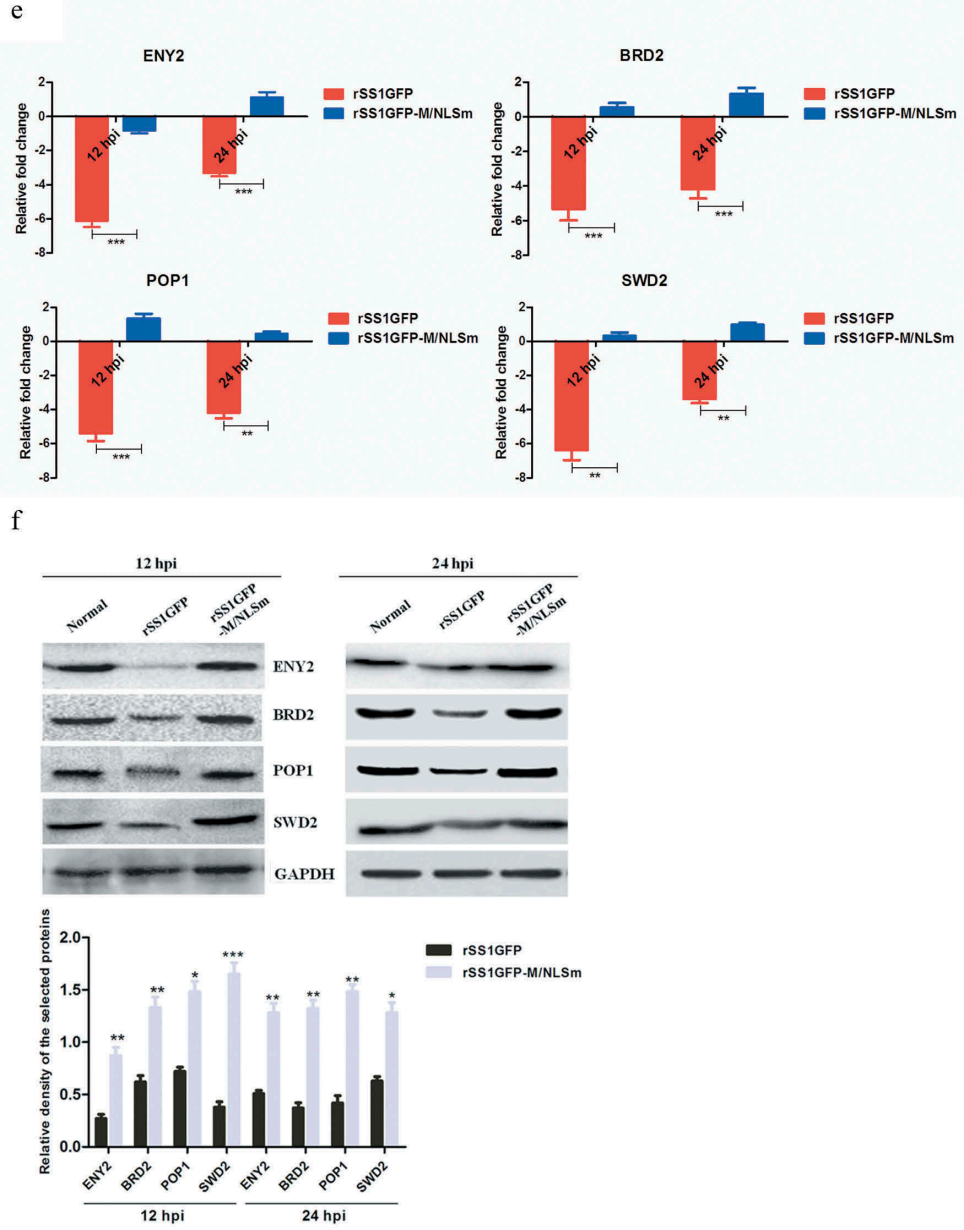
(Continued).

To better understand how NDV interacts with the cellular proteins and affects cell functions, the DEPs were analyzed by searching the String database and the protein-protein interaction network. As shown in Figure 7(b), there was one group of proteins (BRD2-TBP-MYBBP1A-POLR1E-ABT1) participated in “Transcription” process, which strongly interacted and was significantly regulated by rSS1GFP infection. Among them, BRD2 [36], TBP [37], MYBBP1A [38], POLR1E [39], and ABT1 [40] have been demonstrated to regulate cell transcription. In addition, two groups of proteins (POP1-NHP2-NOP58-LSM6-TXNL4A

-NHP2L1, SCAF4-NCBP2-SNRPD1-SF3B4-CDC5 L-ZCCHC17-DDX39A) were found to interact strongly with each other and participated in “RNA processing and modification” process (Figure 7(d)). Meanwhile, further investigation revealed that most of these DEPs in this interaction network are known to play important roles in RNA processing and modification.

To further validate the DEPs identified by TMT-labeled LC-MS/MS analysis, the expression of two DEPs in each category (ENY2 and BRD2 for “Transcription”, POP1 and SWD2 for “RNA processing and modification”) was detected by qRT-PCR and Western blotting, respectively. The results of qRT-PCR showed that the mRNA expression levels of four selected genes in rSS1GFP-infected cells were the lowest at 12 hpi, and were still obviously down-regulated compared with the rSS1GFP-M/NLSm group at 24 hpi (Figure 7(e)). In addition, the expression patterns analyzed by Western blot analysis were consistent with the qRT-PCR results and TMT-labeled LC-MS/MS findings (figure 7(f)). Together with the above results, these results indicated that the dominant nuclear accumulation of NDV M protein could inhibit cell transcription and RNA processing and modification processes.

### rSS1GFP infection affects the expression of cellular translation, posttranslational modification and trafficking-associated proteins

The host protein synthesis and processing machineries can be hijacked and usurped by viruses to synthesize, modify and transport viral proteins and also stifle host innate defense to facilitate viral propagation [41–43]. Therefore, according to the results of COG/KOG categories analysis, the expression profiles of DEPs related to “Translation, ribosomal structure and biogenesis”, “Posttranslational modification, protein turnover, chaperones” and “Intracellular trafficking, secretion, and vesicular transport” were analyzed. A total of 97, 88 and 56 DEPs associated with the three categories were found in rSS1GFP group, respectively, but 20 representative DEPs of each category were selected for further analysis. We found that relatively lower expression profiles of DEPs associated with the three categories were observed in rSS1GFP group compared to that of rSS1GFP-M/NLSm group at 12 hpi (Figure 8(a,c,e)). However, the expression of DEPs related to “Translation, ribosomal structure and biogenesis”, “Posttranslational modification, protein turnover, chaperones” and “Intracellular trafficking, secretion, and vesicular transport” was significantly increased in rSS1GFP group at 24 hpi, which was especially much higher in “Translation, ribosomal structure and biogenesis” category (Figure 8(a,c,e)). As for the rSS1GFP-M/NLSm group, the expression of most DEPs in “Translation, ribosomal structure and biogenesis” and “Posttranslational modification, protein turnover, chaperones” were decreased at 12 hpi, but then increased at 24 hpi (Figure 8(a,c)). By contrast, most of the DEPs in “Intracellular trafficking, secretion, and vesicular transport” showed the expression from up-regulated at 12 hpi to down-regulated at 24 hpi (Figure 8(e)).

**Figure 8.**
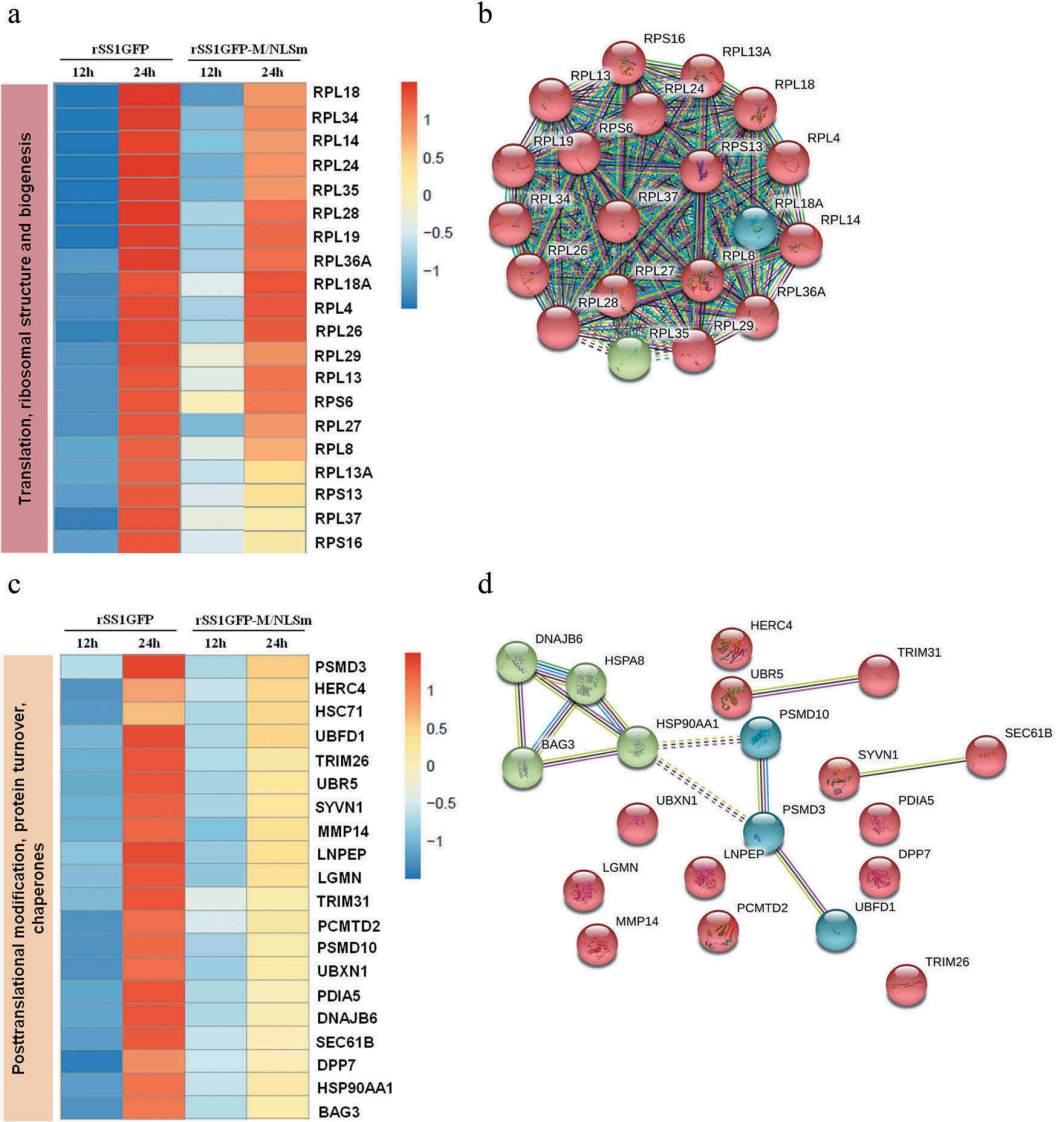
rSS1GFP infection affects the expression of cellular translation, posttranslational modification and trafficking-associated proteins. (a) The heatmap of representative 20 DEPs related to “Translation, ribosomal structure and biogenesis” during rSS1GFP and rSS1GFP-M/NLSm infection at 12 and 24 h. (b) The protein-protein interactions of the DEPs related to “Translation, ribosomal structure and biogenesis” are analyzed by the STRING software. A red line indicates the presence of fusion evidence; a blue line indicates co-occurrence evidence; a light blue line indicates database evidence; a purple line indicates experimental evidence; a green line indicates neighborhood evidence; a black line indicates co-expression evidence. (c) The heatmap of representative 20 DEPs related to “Posttranslational modification, protein turnover, chaperones” during rSS1GFP and rSS1GFP-M/NLSm infection at 12 and 24 h. (d) The protein-protein interactions of the DEPs related to “Posttranslational modification, protein turnover, chaperones” are analyzed by the STRING software. (e) The heatmap of representative 20 DEPs related to “Intracellular trafficking, secretion, and vesicular transport” during rSS1GFP and rSS1GFP-M/NLSm infection at 12 and 24 h. (f) The protein-protein interactions of the DEPs related to “Intracellular trafficking, secretion, and vesicular transport” are analyzed by the STRING software. (g) The mRNA expression levels of six selected DEP genes in BSR-T7/5 cells infected with rSS1GFP and rSS1GFP-M/NLSm were verified by qRT-PCR. (h) The protein expression levels of six DEPs in BSR-T7/5 cells infected with rSS1GFP and rSS1GFP-M/NLSm were examined by Western blotting. The relative expression levels of six DEPs were compared with the control GAPDH expression. Error bars represent standard deviations (mean ± SD) (*P < 0.05; **P < 0.01; ***P < 0.001 compared to the value of rSS1GFP-M/NLSm).

**Figure 8.**
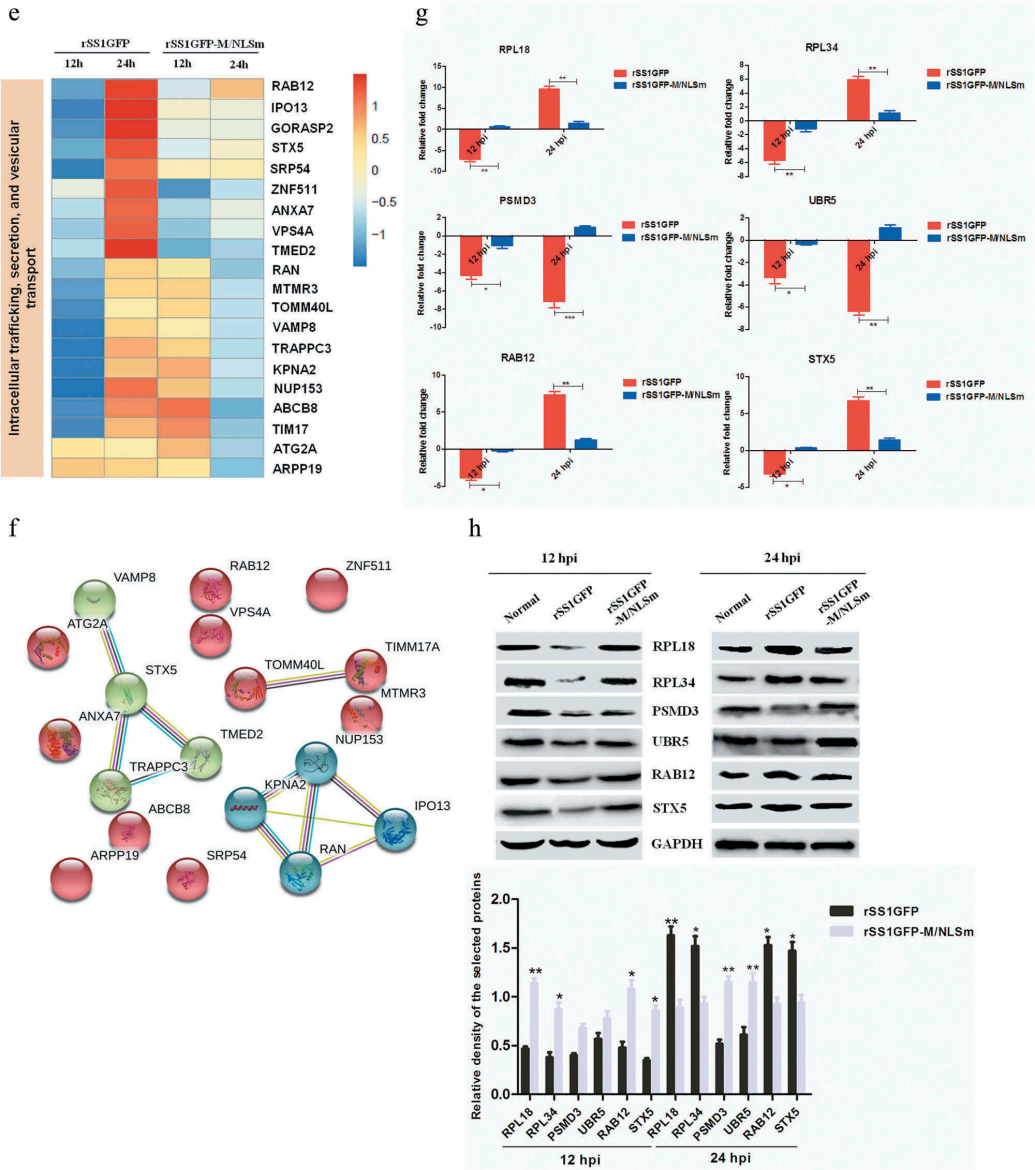
(Continued).

Analysis of the protein-protein interaction networks indicated that complex and strong interactions existed in these ribosomal proteins, which contributed to “Translation, ribosomal structure and biogenesis” process (Figure 8(b)). In addition, two groups of proteins including (BAG3-DNAJB6-HSP90AA1-HSPA8, and PSMD10-PSMD3-UBFD1) and (VAMP8-STX5-TRAPPC3-TMED2, and NUP153-KPNA2-RAN-IPO13) were found to interact strongly with each other and participated in “Posttranslational modification, protein turnover, chaperones” and “Intracellular trafficking, secretion, and vesicular transport” processes, respectively (Figure 8(d,f)). Moreover, literature search revealed that most of these DEPs in the three interaction networks play crucial roles in these biological processes. Next, the expression of two DEPs in each category (RPL18 and RPL34, PSMD3 and UBR5, RAB12 and STX5) was confirmed by qRT-PCR and Western blotting, respectively. The results of mRNA expression of DEP genes detected by qRT-PCR and expression of DEPs detected by Western blotting remained basically consistent (Figure 8(g,h)), which were also consistent with the TMT-labeled LC-MS/MS analysis. Thus, these data suggested that the nuclear aggregation of NDV M protein might participate in interfering with cellular protein synthesis, posttranslational modification and trafficking, but this kind of inhibition function could be weakened when most of the M protein was confined outside the nucleus.

### rSS1GFP replication is enhanced by inhibiting TIFA/TRAF6/NF-κB signaling pathway

It is worth noting that TRAF-interacting protein with FHA domain-containing protein A (TIFA) was one of the jointly owned DEPs between rSS1GFP group and rSS1GFP-M/NLSm group at 12 and 24 hpi (Table 2). However, according to the TMT-labeled LC-MS/MS analysis, TIFA was the highest and the lowest expressed DEP in the two groups at 12 and 24 hpi, respectively (Table 2). Multiple research groups have demonstrated that the interaction between phosphorylated Thr9 of TIFA and tumor necrosis factor receptor-associated factor 6 (TRAF6) is the key mechanism for TIFA-mediated nuclear factor-κB (NF-κB) activation [44–46], which transactivates many cytokines and inflammation-associated transcriptional factors in response to immune response and inflammatory challenges [47,48]. These results indicated that TIFA/TRAF6/NF-κB signaling pathway might play crucial roles in the antiviral immune response. Therefore, we further gained insight into the role of TIFA/TRAF6/NF-κB in NDV replication.

The mRNA expression and protein expression of TIRA in rSS1GFP- and rSS1GFP-M/NLSm-infected cells were first analyzed by qRT-PCR and Western blotting, respectively. As shown in Figure 9(a,b), the quantitative results trends of TIFA in mRNA and protein expression patterns were consistent with the TMT-labeled LC-MS/MS findings. To investigate whether the M protein alone and the subcellular localization of M protein can affect the expression of TIFA, we co-transfected the plasmids pCMV-HA-TIFA and pEGFP-M or pEGFP-M/NLSm into BSR-T7/5 cells, respectively. The fluorescent co-localization results showed that EGFP-M mainly localized in the nucleus and nucleolus, and HA-TIFA was present in the cytoplasm; whereas EGFP-M/NLSm exhibited the clear co-localization with HA-TIFA in the cytoplasm (Figure 9(c)). In addition, transfection of 0.5 μg pEGFP-M or pEGFP-M/NLSm nearly had no effect on the expression of endogenous TIFA, but when the transfection dose of pEGFP-M/NLSm increased, the expression of TIFA was obviously decreased in comparison to the pEGFP-M transfection group (Figure 9(d)). Overall, these results suggested that the M protein in the cytoplasm was able to reduce the expression of TIFA in a dose-dependent manner.

**Figure 9.**
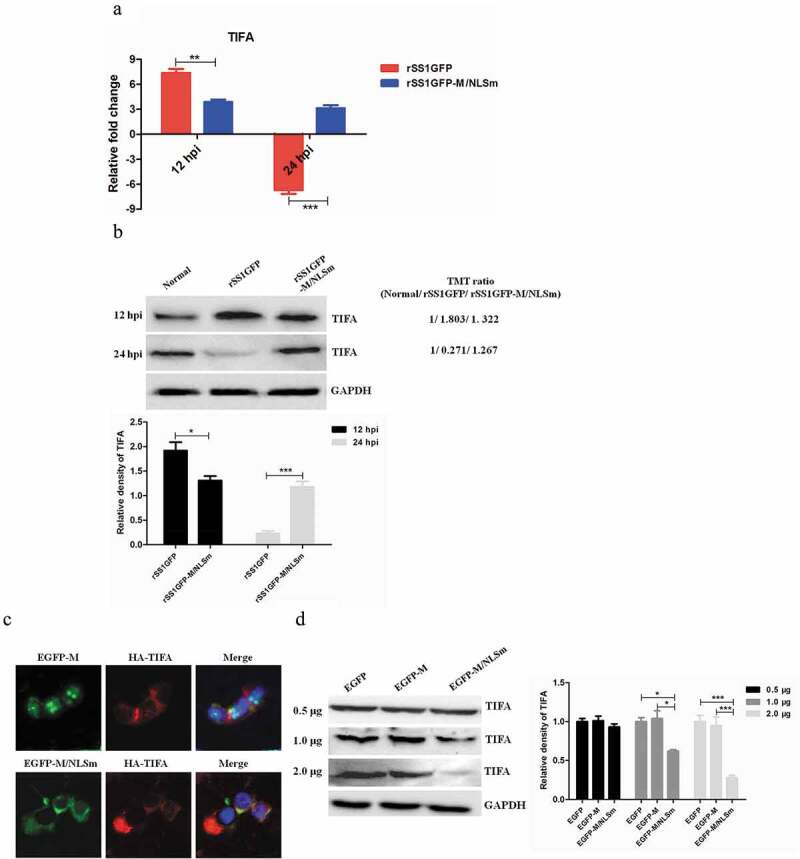
rSS1GFP replication is enhanced by inhibiting TIFA/TRAF6/NF-κB signaling pathway later in infection. (a) The mRNA expression levels of TIFA gene in BSR-T7/5 cells infected with rSS1GFP and rSS1GFP-M/NLSm were verified by qRT-PCR. (b) The protein expression levels of TIFA in BSR-T7/5 cells infected with rSS1GFP and rSS1GFP-M/NLSm were examined by Western blotting. The relative expression levels of TIFA were compared with the control GAPDH expression. (c) The subcellular localization of EGFP-M or EGFP-M/NLSm and HA-TIFA in plasmids co-transfected BSR-T7/5 cells. Original magnification was 1 × 200. (d) The effect of different dosage EGFP-M or EGFP-M/NLSm on the expression level of endogenous TIFA in plasmid transfected BSR-T7/5 cells. The relative expression levels of TIFA were compared with the control GAPDH expression. (e) The expression patterns of TIFA, pTIFA, TRAF6, NF-κB p65, pNF-κB p65, and IL-2 in BSR-T7/5 cells infected with rSS1GFP and rSS1GFP-M/NLSm at 12 and 24 hpi. The relative expression levels of these proteins were compared with the control GAPDH expression. (f) The effect of TIFA overexpression on the expression of IL-2 and virus titers of rSS1GFP and rSS1GFP-M/NLSm at 12 and 24 hpi. (g) The effect of siRNA-mediated knockdown of TIFA on the expression of IL-2 and virus titers of rSS1GFP and rSS1GFP-M/NLSm at 12 and 24 hpi. (h) The schematic diagram illustrated that the M protein in the cytoplasm inhibited host cell immune response by down-regulating TIFA/TRAF6/NF-κB signaling pathway.

**Figure 9.**
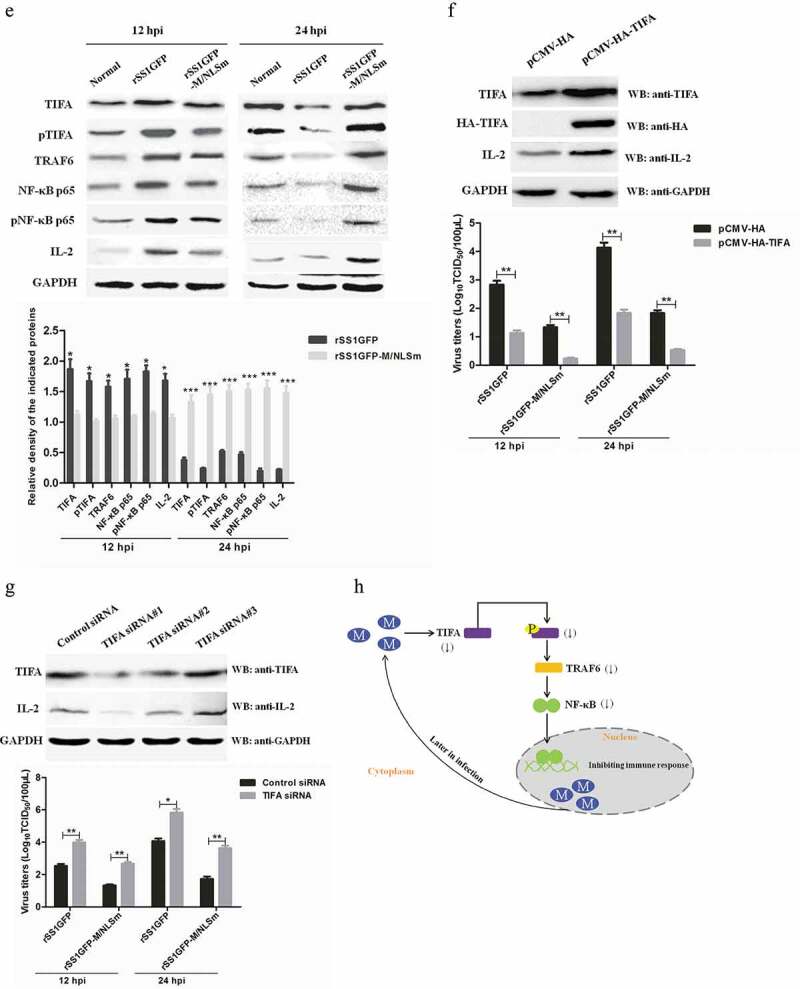
(Continued).

The expression changes of TIFA, phosphorylated TIFA (pTIFA), TRAF6, NF-κB p65, phosphorylated NF-κB p65 (pNF-κB p65) and its transactivated downstream protein IL-2 were then analyzed in rSS1GFP- and rSS1GFP-M/NLSm-infected cells. We found that the expression patterns of these proteins were increased during the course of rSS1GFP and rSS1GFP-M/NLSm infection at 12 hpi, but rSS1GFP infection caused much higher increase than rSS1GFP-M/NLSm infection (Figure 9(e)). However, in contrast to the up-regulated expression of these proteins in rSS1GFP-M/NLSm infection at 24 hpi, rSS1GFP infection remarkably caused the decreased expression of these proteins (Figure 9(e)). In addition, plasmid pCMV-HA-TIFA was transfected into BSR-T7/5 cells to perform the overexpression of HA-TIFA, but the overexpressed HA-TIFA greatly increased the expression of IL-2 and reduced the virus titers of rSS1GFP and rSS1GFP-M/NLSm in virus-infected cells at 12 and 24 hpi (figure 9(f)). Moreover, small interfering RNA (siRNA)-mediated knockdown of TIFA (TIFA siRNA#1) in BSR-T7/5 cells obviously reduced the expression of IL-2 and increased the replication efficiency of rSS1GFP and rSS1GFP-M/NLSm at 12 and 24 hpi (Figure 9(g)). Together with the above the results, these data demonstrated that the cytoplasmic localization of M protein was able to reduce the expression of TIFA and facilitate NDV replication by down-regulating the TIFA/TRAF6/NF-κB-mediated production of cytokines (Figure 9(h)).

## Discussion

Viruses have co-evolved with their hosts for many years and developed effective approaches for hijacking and manipulating host cellular processes [41]. One of the important processes is that viruses take advantage of specific localization of viral proteins, which dynamically and temporally regulates their interactions with host proteins to ensure virus replication and proliferation [13,49]. In recent years, increasing numbers of studies have reported that paramyxoviruses M proteins interact with cellular proteins at special orientation to achieve virus replication. For example, the NDV M protein localizes to the nucleolus through an interaction with B23, and knockdown of B23 results in the reduced CPE and virus replication [50]. The M protein of NiV and HeV transiently interacts with the beta subunit of the AP-3 adapter protein complex AP3B1 at the plasma membrane, which is effective for promoting viral particle assembly [51]. In addition, a recent study has demonstrated that annexin A2 mediates the localization of MeV M protein at the plasma membrane by interacting with its N-terminal region, thereby aiding in MeV assembly [52]. Up to now, the presence of intrinsic NLS and NES within M protein for its nucleocytoplasmic trafficking have been reported in most of the paramyxoviruses [53]. We previously showed that the replication and pathogenicity of NDV is significantly attenuated by M/NLS mutation [10] and the recombinant NDV carrying mutated M/NESs cannot be rescued [11]. In this study, we also found that the disruption of M’s nucleocytoplasmic trafficking by mutating M/NLS remarkably reduced the proliferative ability and cytopathogenicity of NDV. All of these findings suggested that the replication and pathogenicity of NDV was tightly associated with the nucleocytoplasmic trafficking of M protein. However, the attenuated replication mechanism of NDV caused by M/NLS mutation and the potential functions of M’s nucleocytoplasmic trafficking still remain enigmatic.

Viral RNA synthesis, transcription and translation are the key steps in the process of NNSVs replication [2,54]. Although the M protein of NNSVs is known to play key roles in virus assembly later in infection [1,12], based on the findings from HRSV, VSV and SeV, the M protein also modulates viral RNA synthesis and can inhibit the transcriptase activity through M-NP interaction early in infection, thereby repressing the signal to switch from transcription to packaging into the virion particles [12,55,56]. The relevant evidence is that the SeV M protein is cross-linked to the NP protein in generated progeny virions [57], and the addition of M protein to SeV and VSV nucleocapsids decreases their ability to transcribe viral RNA [55,58]. In addition, RNA interference with the M gene efficiently increases viral transcription levels in MeV-infected cells [59], and in minigenome reporter gene assays, the M protein of MeV inhibits viral RNA synthesis only when it interacts with the NP protein [60]. The NDV M protein is reported to be necessary and sufficient for virus budding, and the M-HN and M-NP interactions are responsible for the incorporation of HN and NP proteins into virion particles [5]. Interestingly, we similarly found that viral RNA synthesis and transcription efficiency were greatly decreased by M/NLS mutation in rSS1GFP-M/NLSm-infected cells, suggesting that precocious cytoplasmic localization of M protein had negative effects on viral RNA synthesis and transcription. Because the NNSVs M proteins can avoid the inhibition of viral transcriptase activity early in infection and mediate the association of the nucleocapsid with the nascent viral envelope later in infection, thus we concluded that the dominant nuclear accumulation of NDV M protein might ensure that viral RNA synthesis and transcription in the cytoplasm proceeded smoothly until a certain level of viral RNA and protein expression was synthesized, at which point the M protein entered the cytoplasm and plasma membrane to achieve virus assembly and budding. Therefore, the reduced viral RNA synthesis and transcription caused by M/NLS mutation might be one of the reasons responsible for the attenuated replication of rSS1GFP-M/NLSm.

Virus-host protein interactions based on quantitative proteomics analysis have become important methods in understanding cellular proteins involved in virus replication and pathogenesis [22,23,41,42]. In this study, the quantitative TMT and LC-MS/MS approach were applied to compare the proteome of BSR-T7/5 cells in response to rSS1GFP and rSS1GFP-M/NLSm infection. We found that the two viruses showed great discrepancy in terms of stimulating host protein expression profiles (484 vs 109 DEPs and 466 vs 104 DEPs between rSS1GFP and rSS1GFP-M/NLSm at 12 and 24 hpi, respectively), and only seven DEPs were shared by these two viruses. GO enrichment analysis of the DEPs showed that most of the DEPs were functionally related to the structural constituent of ribosome and structural molecule activity in rSS1GFP group and rSS1GFP-M/NLSm group, but rSS1GFP infection caused more changes in GO enrichment. The KEGG pathway analysis further determined that these DEPs were mainly involved in important cellular pathways including ribosome, Glycolysis/Gluconeogenesis, lysosome signaling pathways in rSS1GFP group. In addition, further COG/KOG categories analysis revealed that rSS1GFP virus highly activated more DEPs to participate in “Translation, ribosomal structure and biogenesis”, “Signal transduction mechanisms”, “Posttranslational modification, protein turnover, chaperones”, “Intracellular trafficking, secretion, and vesicular transport”, “Transcription”, “RNA processing and modification”, “Cytoskeleton” and so on. Therefore, these results suggested that rSS1GFP infection could cause much more expression changes of DEPs, which were involved in numerous functional categories and KEGG pathways, to assist virus replication in comparison to rSS1GFP-M/NLSm infection.

Numerous studies have demonstrated that M’s nuclear localization of NNSVs such as HRSV [7], MeV [9], and VSV [61,62] has the capability to inhibit host cell transcription independently of other viral components. Supporting this conclusion is the fact that nuclear extracts from HRSV-infected cells have less transcriptional activity *in vitro* and also inhibit the transcriptional activity of nuclear extracts from mock-infected cells [7]. In addition, a recent study revealed that transient expression of MeV M protein in plasmid-transfected cells binds to nuclear factors and is able to inhibit *in vitro* transcription in a dose-dependent manner [9]. Additionally, studies focusing on VSV M protein showed that the M protein directly inhibits host cell transcription by inactivating host RNA polymerases Ⅰ and Ⅱ [62], and also interacts with nuclear pore complexes to impair nuclear export of cellular mRNAs [63], thereby indirectly leading to a decrease and an increase in host cell and virus transcription [64,65]. More recently, using microarray analysis of rSS1GFP-infected chicken embryo fibroblasts, we found that nuclear localization of NDV M protein might inhibit host cell transcription, showing that the transcription repressor activity- and negative regulation of transcription-related genes were up-regulated, while the RNA polymerase Ⅱ transcription factor activity- and transcriptional activator activity-related genes were down-regulated [65]. In addition, siRNA-mediated knockdown of the representative up-regulated gene or down-regulated gene significantly reduced or increased viral RNA synthesis and virus replication, respectively [66]. Consistent with this finding, the quantitative proteomics analysis also revealed that most of these DEPs, which had important functions in the process of “Transcription” and “RNA processing and modification” in rSS1GFP infection, exhibited obviously down-regulated expression patterns. Meanwhile, it is notable that in addition to the transcription activation function, both ENY2 and THOC2 are also demonstrated to be involved in cellular mRNA nuclear export [67,68]. However, whether the NDV M protein can interact with ENY2 or THOC2 to impair nuclear export of cellular mRNAs remains to be explored. Remarkably, several DEPs associated with RNA transport, RNA degradation, RNA polymerase, basal transcription factors, spliceosome, mRNA surveillance pathways in rSS1GFP group showed much more and lower expression levels than that in rSS1GFP-M/NLSm group at 12 and 24 hpi (Supplemental material Table S7). Based on these findings, we speculated that the inhibition of host cell transcription caused by the dominant nuclear accumulation of NDV M protein might occur through more diverse pathways than that of other NNSVs. Together, these results indicated that the weakened inhibition of host cell transcription due to M/NLS mutation possibly affected viral transcription and replication, which might be another reason responsible for the attenuated replication of rSS1GFP-M/NLSm.

Ribosomal proteins (RPs) are the major components of ribosomes involved in the cellular process of protein biosynthesis. Nowadays, increasing evidence has demonstrated that multifarious RPs interact with viral proteins to participate in viral protein biosynthesis and regulate virus replication in host cells [69]. For example, 60S ribosomal protein L18 (RPL18) is found to be incorporated into Ebola virions, and the reduced expression of RPL18 effectively represses Ebola virus infection [70]. Rice stripe tenuivirus nucleocapsid protein interacts with RPL18 of insect vector and silencing of RPL18 obviously reduces viral translation and replication [71]. In addition, RNA interference of ribosomal protein RPL34 causes serious damages to abortive infection of Autographa californica multiple nucleopolyhedrovirus, indicating that ribosomal components are essential for productive baculovirus infection [72]. Meanwhile, recently relevant research results also suggested that many other RPs including RPL24, RPL19, RPL4, RPS6 and so on play critical roles in the life cycle of viruses. In our studies, we found that most of the RPs showed significantly down-regulated in rSS1GFP group at 12 hpi, but exhibited remarkably up-regulated at 24 hpi when compared to rSS1GFP-M/NLSm group. This kind of changing trends of RPs was also clearly observed in the hierarchical clustering heat map of ribosome-related DEPs and the modeling of ribosome signaling pathway in the two viruses-infected cells at 12 and 24 hpi. A previous study has demonstrated that NDV is the most effective paramyxovirus at inhibiting the production of host proteins [73]. One possible explanation for this was due to the inhibition of host cell transcripts and the drastic reduction of RPs early in NDV infection. It is noteworthy that RPL18 has been reported to interact with multiple viral proteins and participate in viral protein biosynthesis [69]. Here, RPL18 was the highest up-regulated DEP during rSS1GFP infection at 24 hpi (rSS1GFP, 4.169-fold up-regulation; rSS1GFP-M/NLSm, 1.286-fold up-regulation), and the viral proteins (such as NP and M) were increasingly expressed in rSS1GFP group at 24 hpi, indicating that RPL18 played crucial roles in promoting viral protein biosynthesis. However, further studies are necessary to investigate whether NDV M protein interacts with RPL18 and how this interaction regulates NDV viral protein biosynthesis. Overall, these results suggested that the dominant nuclear aggregation of M protein was conducive to inhibit the expression of cellular RPs, and viral protein synthesis could be enhanced when most of the M protein entered the cytoplasm.

Currently, accumulating studies have indicated that viruses employ various strategies to hijack and usurp host cellular machinery for their own benefit [41,42,74]. The representative studies revealed that many identified abundant proteins in IAV-infected cells are associated with protein synthesis, chaperone-mediated responses, protein metabolism and posttranslational modification, including protein folding, proteolysis, and the ubiquitin-proteasome system [75–77]. In this study, according to “Posttranslational modification, protein turnover, chaperones” analysis in rSS1GFP group, we found that in addition to the few DEPs related to proteasome and chaperones, most DEPs belonged to ubiquitin-protein ligases such as HERC4, UBFD1, TRIM26, TRIM31, UBR5, UBXN1 and so on. Importantly, these DEPs showed obviously down-regulated at 12 hpi, but exhibited remarkably up-regulated at 24 hpi during rSS1GFP infection. Studies have shown that angiomotin-like 1 is a PIV5 M-interacting protein and serves as a linker between paramyxovirus budding and the ESCRT pathway [78]. The subsequent study found that angiomotin-like 1 links PIV5 and MuV M proteins to NEDD4 family ubiquitin ligases, which represents a novel host factor recruitment strategy for paramyxoviruses to achieve viral particle release [79]. In addition, another study also demonstrated that ubiquitin-regulated nucleocytoplasmic trafficking of the NiV M protein is associated with M’s post-translational modification and plays important roles in M-mediated viral budding [21]. These results suggested that ubiquitin ligases might participate in the nucleocytoplasmic trafficking of M protein and the assembly and budding of NDV progeny virions, but whether NDV M protein interacts with some of these ubiquitin ligases-related DEPs and the exact role of their interaction in NDV budding are worth to be deeply studied. Interestingly, rSS1GFP infection also changed the expression patterns of DEPs involved in “Intracellular trafficking, secretion, and vesicular transport”. The possible explanation for these changing trends might be that some NDV proteins such as NP, P, F, and HN interfered with the expression of DEPs in endoplasmic reticulum during the nuclear localization of M protein [80,81], but the interference effect was weakened when the M protein entered the cytoplasm to interact with these viral proteins. It is remarkable that many enveloped viruses including NDV encode late domain motifs that are able to hijack VPS4A and/or VPS4B to complete viral budding [11,82]. Compared to the expression level of VPS4A in rSS1GFP-M/NLSm group, VPS4A showed highly up-regulated expression pattern in rSS1GFP group at 24 hpi, suggesting that rSS1GFP possessed much stronger budding ability than rSS1GFP-M/NLSm. Meanwhile, some DEPs such as STX5, KPNA2, IPO13, RAN, NUP153, which showed up-regulated expression in rSS1GFP group, are associated with intracellular trafficking and vesicular transport and have been reported to regulate virus replication. Therefore, together with the above results, we speculated that the relatively decreased expression of DEPs involved in ribosome structure, protein posttranslational modification and trafficking due to the disrupted nuclear accumulation of M protein affected viral protein synthesis and budding, which might be the third reason responsible for the attenuated replication of rSS1GFP-M/NLSm.

Inflammatory responses are important aspects of the innate immune system during virus infection. In previous studies, several signaling pathways, such as IRAK1/TRAF6/NF-κB, TLR4/TRAF6/NF-κB, SIRT1/AMPK/NF-κB, VEGFA/ERK1/2/NF-κB and so on, have been reported to participate in inflammatory responses. Recently, alpha-kinase 1 (ALPK1) controlling TIFA/TRAF6-dependent innate immunity against bacterial infection is gradually reported [48,83–85]. It has been demonstrated that TIFA serves as a TRAF6 binding protein and plays a major role in the activation of NF-κB [44,46,48], which transactivates various of cytokines, chemokines, and inflammation-associated transcriptional factors in response to immune response and inflammatory challenges. However, the role of TIFA/TRAF6/NF-κB signaling pathway in the replication of viruses remains unknown. In our studies, we found that the protein expression of TIFA in rSS1GFP group was high up-regulation at 12 hpi, but showed very low down-regulation at 24 hpi when compared to that in rSS1GFP-M/NLSm group. The quantitative results of TIFA in mRNA and protein expression patterns detected by qRT-PCR and Western blotting were consistent with the TMT-labeled LC-MS/MS analysis. In addition, we also found that the M protein in the cytoplasm effected the inhibition of TIFA expression in a dose-dependent manner. Moreover, the expression of TIFA, pTIFA, TRAF6, NF-κB, pNF-κB and IL-2 was increased at 12 hpi, but decreased at 24 hpi in rSS1GFP-infected cells. Furthermore, overexpression of TIFA or siRNA-mediated knockdown of TIFA obtained the different results, which showed the obviously increased or decreased expression of IL-2 and the remarkably raised or reduced virus titers in rSS1GFP- and rSS1GFP-M/NLSm-infected cells. It is reported that virulent NDV infection induces transcriptional up-regulation of numerous cytokines, such as IFN-α, IFN-γ, IL-8, IL-2, and IL-1β [86,87]. However, overexpression of IL-2 or IL-1β can lead to the decreased systemic viral load and pathogenicity of virulent NDV [88,89]. Thus, these results indicated that the inhibition of TIFA expression could reduce NF-κB-mediated production of cytokines by down-regulating TIFA/TRAF6/NF-κB signaling pathway, which was beneficial to NDV replication. While the precocious cytoplasmic localization of M protein could not down-regulate TIFA/TRAF6/NF-κB signaling pathway, which might be the fourth reason responsible for the attenuated replication of rSS1GFP-M/NLSm.

**Figure 10.**
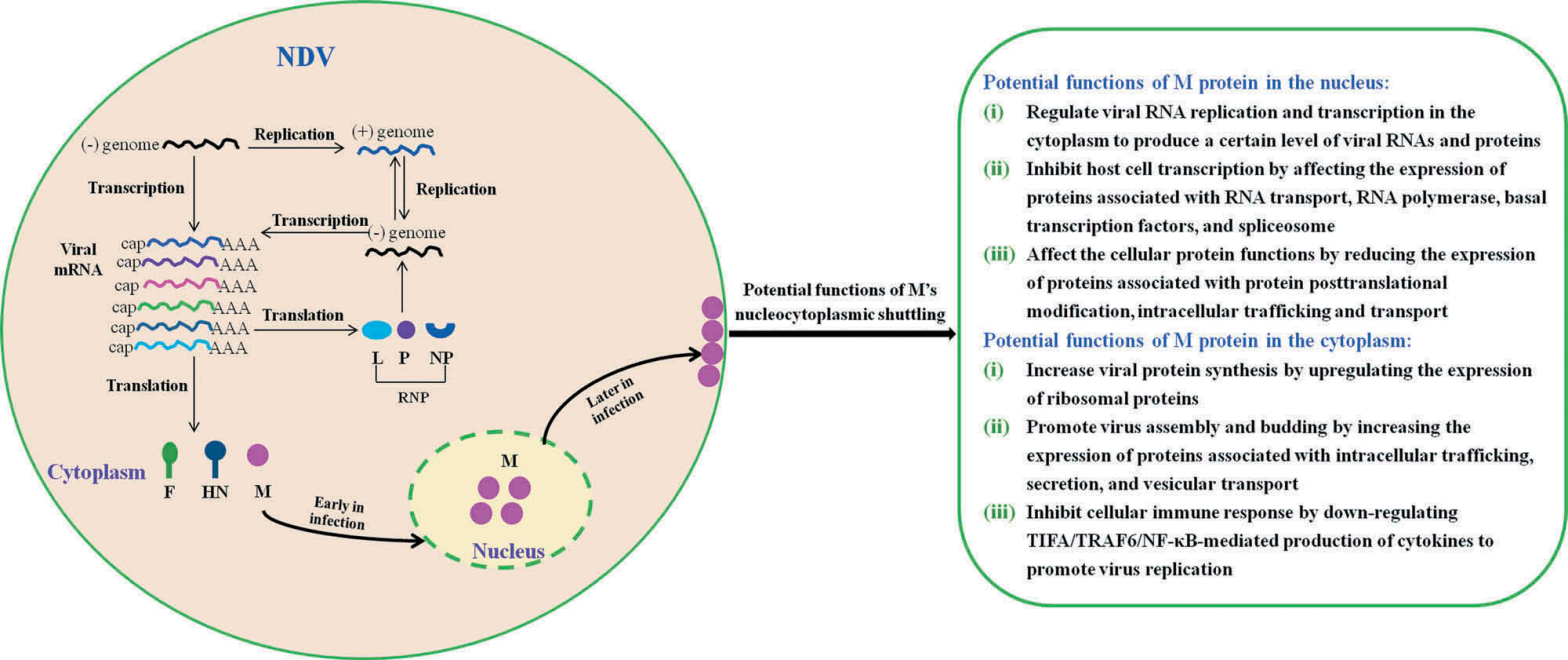
The schematic diagram of the potential functions of M’s nucleocytoplasmic trafficking. Replication and transcription of NDV genome occurs in the cytoplasm via the action of viral ribonucleoprotein (RNP) complexes. During the course of NDV infection, the M protein localizes to the nucleus early in infection and enters the cytoplasm and binds to the cellular plasma membrane later in infection. The potential functions of M protein in the nucleus and the cytoplasm are indicated according to our findings.

In summary, we demonstrated that nucleocytoplasmic trafficking of M protein played crucial roles in regulating viral RNA synthesis and transcription, and participating in the hijack and despoil of host cellular machinery for NDV replication (Figure 10). However, M/NLS mutation disrupted the nucleocytoplasmic trafficking of M protein and affected these important biological processes, which in turn caused the attenuated replication of NDV. Our findings therefore reveal for the first time that the replication of NDV is closely associated with the nucleocytoplasmic trafficking of M protein, which provides a better understanding of the potential functions of M’s nucleocytoplasmic trafficking in NDV life cycle and also aids in understanding the poorly understood molecular pathogenesis of NDV.

## Materials and methods

### Cells, viruses and antibodies

BSR-T7/5 cells stably expressing the T7 phage RNA polymerase were generated by Buchholz et al. [90], and were a kind gift from Prof. Xiufan Liu (Key Laboratory of Animal Infectious Diseases, Yangzhou University, China). The parental NDV (rSS1GFP) and the mutant NDV (rSS1GFP-M/NLSm) carrying M/NLS mutation were generated in our previous study [10], which were plaque purified three times in chicken embryonic fibroblasts and propagated once in specific pathogen-free (SPF) embryonated chicken eggs. The rabbit polyclonal antibodies against NDV M protein and NP protein was prepared in our laboratory. The rabbit polyclonal antibodies against ENY2 (DF12979), BRD2 (DF12857), RPL18 (DF3700), RPL34 (DF3708), PSMD3 (DF3645), RAB12 (DF12459), TRAF6 (AF5376), NF-κB p65 (AF5006), phospho-NF-κB p65 (Ser536) (AF2006), GAPDH (AF7021), GFP (T0006), HA (T0050) and IL-2 (AF5105) were purchased from Affinity Biosciences (USA). The rabbit polyclonal antibodies against POP1 (ab254978), SWD2 (ab220240), UBR5 (ab70311), STX5 (ab217130), and rabbit monoclonal antibody against phospho-TIFA (Thr9) (ab214815) were purchased from Abcam (USA). The rabbit polyclonal antibody against TIFA (61358S) was purchased from Cell Signaling Technology (USA).

### Cell culture and Virus infection

BSR-T7/5 cells were maintained in Dulbecco’s modified Eagle’s medium (DMEM) supplemented with 10% fetal bovine serum (FBS) and were cultured at 37°C under 5% CO_2_. BSR-T7/5 cells grown in 6-well plates were infected with NDV strain rSS1GFP or rSS1GFP-M/NLSm at a multiplicity of infection (MOI) of 1. The cell culture supernatants were collected at different time points (6, 12, 24, 36, 48, 72, and 96 hpi), and the virus titers were determined as 50% tissue culture infective dose (TCID_50_) in BSR-T7/5 cells. In addition, the CPE and green fluorescence in virus-infected cells were observed under a inverted fluorescence microscope (Nikon, Japan) and the expression levels of NP, M and GFP were detected by Western blot at 6, 12 and 24 hpi, respectively. Moreover, the brightness of green fluorescence in viruses-infected cells was quantitatively compared by Image J software version 1.8.0.

### Indirect immunofluorescence assay

BSR-T7/5 cells cultured in 12-well plates were infected with NDV strain rSS1GFP or rSS1GFP-M/NLSm at an MOI of 1 and prepared for indirect immunofluorescence analysis at 6, 12, 18 and 24 hpi, respectively. Briefly, cells were collected at the stipulated times and rinsed thrice with phosphate-buffered saline (PBS), fixed with 4% paraformaldehyde for 20 min at room temperature, and then permeabilized with 0.25% Triton X-100 in PBS for 5 min. Cells were rinsed thrice with PBS and blocked with 10% FBS in PBS for 1 h, and then incubated with rabbit anti-M polyclonal antibody diluted in PBS containing 10% FBS for 1 h at 37°C. After three washes with PBS, the cells were incubated with Cy3-labeled goat anti-rabbit IgG (H + L) (Beyotime Biotechnology, China) for 1 h at 37°C. Cells were rinsed thrice with PBS and then counterstained with DAPI (Sigma) to detect the nuclei. Images were captured with a fluorescence microscope and processed with Adobe Photoshop CS5 software.

### Quantification of viral RNA synthesis and gene expression by qRT‑PCR

BSR-T7/5 cells grown in 6-well plates were infected with rSS1GFP or rSS1GFP-M/NLSm at an MOI of 1. Cells were collected at the indicated time points (6, 12, and 24 hpi), and total RNA was extracted using TRIzol reagent (Invitrogen) according to the manufacturer’s instructions. The resulting RNA samples (2 μg per sample) were reverse-transcribed as previously described [91]. Quantification of viral RNA synthesis (the RNA levels of NP and P genes) and gene expression (the mRNA levels of M and GFP genes) by qRT-PCR was performed using previously reported methods [66,92]. The relative gene expression levels were normalized to that of the GAPDH gene. The threshold cycle 2^−ΔΔCT^ method was used to determine the fold change of gene expression levels.

### Protein sample preparation and trypsin digestion

Three biological replicates of two virus-infected groups and one mock group were prepared for TMT-based quantitative proteomics experiments (Figure 2). Briefly, BSR-T7/5 cells grown in 25-cm^2^ flasks were infected with rSS1GFP or rSS1GFP-M/NLSm at an MOI of 1. Cell samples were collected at 12 and 24 hpi, respectively, and then sonicated three times on ice using a high intensity ultrasonic processor (Scientz) in lysis buffer (8 M urea, 1% protease inhibitor cocktail). The remaining debris was removed by centrifugation at 13,000 g at 4°C for 10 min. The supernatant was collected and the protein concentration was determined using the BCA kit (Beyotime Biotechnology, China) according to the manufacturer’s instructions. For trypsin digestion, the protein solution was reduced with 5 mM DTT for 30 min at 56°C and alkylated with 11 mM iodoacetamide for 15 min at room temperature. The protein sample was then diluted by adding 100 mM TEAB to urea concentration less than 2 M. Finally, trypsin was added at 1:50 (trypsin: protein) mass ratio for the first digestion overnight and 1:100 (trypsin: protein) mass ratio for a second 4 h-digestion.

### TMT labeling, HPLC fractionation and LC-MS/MS analysis

After trypsin digestion of protein samples, peptide was desalted by Strata X C18 SPE column (Phenomenex) and vacuum-dried. Peptide was reconstituted in 0.5 M TEAB and processed for 6-plex TMT kit according to the manufacturer’s instructions. Briefly, one unit of TMT reagent was thawed and reconstituted in acetonitrile (about 100 mg protein). 41 μL of TMT Label Reagent was carefully added to each 100 μL sample, and eighteen samples were differentially labeled with six TMT tags (Control group: 126 label for 12 h and 129 label for 24 h; rSS1GFP group: 127 label for 12 h and 130 label for 24 h; rSS1GFP-M/NLSm group: 128 label for 12 h and 131 label for 24 h). The peptide mixtures were then incubated for 2 h at room temperature. Then reactions were treated with 8 μL 5% hydroxylamine for 15 min to stop trypsin. Finally, the six labeled peptides were combined in a new microcentrifuge tube and 1 mL mixed peptides were dried by vacuum concentrator.

The labeled peptides were fractionated into 60 fractions by high pH reverse-phase high-performance liquid chromatography (HPLC) using Agilent 300Extend C-18 column (5 μm particle size, 4.6 mm ID, 250 mm length) with a gradient of 8% to 32% acetonitrile (pH 9.0) over 60 min. Then, the 60 fractions were combined into 18 fractions and each fraction (volume of 800 μL) was dried by vacuum centrifuging pending for MS analysis. The LC-MS/MS analysis of the labeled peptides was performed as described previously [93]. The related technical support and data analysis were supported by Jingjie PTM BioLabs (Hangzhou, China).

### Database search and data analysis

The resulting MS/MS data were processed using MaxQuant with an integrated Andromeda search engine (v.1.5.2.8). Tandem mass spectra were searched against the UniProt *Cricetulus griseus* database (*Cricetulus_griseus*_uniprot_10029, 23885 sequences) concatenated with reverse decoy database. Common contamination database was added to eliminate the influence of contaminated proteins in the identified proteins. Trypsin/P was specified as the cleavage enzyme allowing up to 2 missing cleavages. The mass tolerance for precursor ions was set as 20 ppm in First search and 5 ppm in Main search, and the mass tolerance for fragment ions was set as 0.02 Da. Carbamidomethyl on Cys was specified as fixed modification and acetylation modification and oxidation on Met were specified as variable modifications. Gene ontology (GO) annotation was derived from the UniProt-GOA database (http://www.ebi.ac.uk/GOA/). Biological processes, cellular components, molecular functions, and KEGG pathways analysis of the DEPs were conducted using DAVID (Database for Annotation, Visualization, and Integrated Discovery) version 6.7. The protein-protein interactions were analyzed by STRING software version 11.0 (http://string.embl.de/).

For TMT quantification, the ratios of the TMT reporter ion intensities in the MS/MS spectra (*m/z* 126–131) from rawdatasets were used to calculate fold changes between samples. False discovery rate (FDR) was adjusted to <1% at protein, peptide and peptide-spectrum match level, and minimum score for peptides was set at >40. Only peptides unique for a given protein were considered for relative quantification. For each sample, the quantification was normalized using the average ratio of all the unique peptides. The two-tailed Fisher’s exact test was employed to test the enrichment of the DEPs versus all identified proteins. For data analysis, *p*-values were adjusted for multiple hypotheses testing based on the Benjamini-Hochberg FDR method, and protein quantification data with *p*-value <0.05 and fold change of >1.2 or <0.83 and was considered as significantly up-regulated or down-regulated proteins. The mass spectrometry proteomics data have been deposited to the ProteomeXchange Consortium via the PRIDE [94] partner repository with the dataset identifier PXD018098.

### Analysis of cellular gene expression by qRT-PCR

Based on the proteomics results, qRT-PCR was used to analyze the expression of 11 selected DEP genes. The primers (Table 3) for qRT-PCR were designed based on the target sequences using Primer Premier 5.0 software. Total RNA was isolated from virus-infected cells or normal cells using the TRIzol reagent according to the manufacturer’s protocol. One microgram of total RNA per sample was reverse-transcribed into cDNA using superscript Ⅳ reverse transcriptase (ThermoFisher Scientific, USA). The qRT-PCR was performed using SYBR® Premix Ex Taq^TM^ⅡKit (Takara Biomedical Technology, China). All of the reactions were performed in a 25 μL volume containing 12.5 μL of 2× SYBR® Premix Ex Taq^TM^Ⅱ, 400 nM of each primer, 1.0 μL ROX Reference DyeⅡ, and 2.0 μL cDNA. The cycling parameters were 1 cycle at 95°C for 30 s followed by 40 cycles at 95°C for 5 s and 60°C for 34 s. One cycle of melting curve analysis was added for all reactions to verify product specificity. The relative gene expression levels were normalized to that of the GAPDH gene. The threshold cycle 2^−ΔΔCT^ method was used to determine the fold change of gene expression levels.

**Table 3.** Quantitative real-time PCR primers used in this study.

Gene name	Forward primer (5ʹ→3ʹ)	Reverse primer (5ʹ→3ʹ)	EMBL No.
ENY2	ATCTCACAATGCATGTCGATGTCC	GAGGCGTTCTCTTTCTCCAGTTTCT	EGW09203
BRD2	GCGAAAGCTCGGACTCTGAGGAA	TGGGATAGGGCAGCCAGTTGTTC	EGW09104
POP1	ACCATGATCTGTGTCCCATCCG	GTATCAGGCTCTTAAATGGGTCGC	EGW04063
SWD2	GGACCATTTGCAACCTTTAAGATGC	GCCGTTGGTGGAAATGAGTATGAGT	EGW05670
RPL18	GTCCCGGATGATCCGAAAAATGA	CTTCAGCTTGGGCACTTCGAGAA	EGW03967
RPL34	ATTTACTTGCGGGGATGCTGCTT	GGTAAACAATCCTGTTGCCAGGG	EGW10449
PSMD3	CCAGGATGTGGAGATGAAAGAGGA	CCAAGGTGACAGTGTCCAGCTCTC	EGW15211
UBR5	GTTTACTTCTGGACATCAAGCCCGT	GGTGTTGGTCGTCTGGTGGTCTTA	EGW09566
RAB12	AAGTATGCTTCGGAAGATGCTGAGC	AACCGCATCCCAGTTATCTGCTGT	EGW05159
STX5	GCAGAGCCGTCAGAATGGAATCCAA	TTCCAATGCGCTTGGCCATAAG	EGW04472
TIFA	CAGGTTTCCCGAGTTCAGTTTGC	TTGAGGTAGCCAAGCTCCTGATTG	EGW12881
GAPDH	TCAAGAAGGTGGTGAAGCAGGCAT	CATCAAAGGTGGAAGAGTGGGAGTC	CAA36368

### Western blotting

BSR-T7/5 cells grown in 6-well plates were infected with rSS1GFP or rSS1GFP-M/NLSm at an MOI of 1. At 12 and 24 hpi, cells were washed thrice with PBS and then lysed with 1× RIPA buffer (Beyotime Biotechnology, China) for total protein extraction. Equivalent amounts of cell lysate (30 μg) were dealt with SDS-PAGE loading buffer, resolved by SDS-PAGE and then transferred electrophoretically onto polyvinylidene fluoride (PVDF) membrane. The membranes were blocked for 1 h at 37°C with 5% nonfat dry milk in Tris-buffered saline with Tween (TBST) buffer and then incubated overnight at 4°C with the primary antibodies against the target DEPs. The blots were washed thrice in TBST buffer and incubated for 1 h at 37°C with horseradish peroxidase-conjugated anti-mouse or rabbit IgG(H + L). Rabbit anti-GAPDH polyclonal antibody was used as an internal standard. The relative levels of the selected proteins to control GAPDH expression were determined by densitometry using Image J software version 1.8.0.

### TIFA overexpression and virus infection

To investigate the effect of TIFA overexpression on the replication of NDV, the constructed plasmid pCMV-HA-TIFA or empty vector control pCMV-HA was transfected into BSR-T7/5 cells using FuGENE® HD transfection reagent (Promega, USA) according to the manufacturer’s instructions. After 36 hours post-transfection (hpt), the expression of HA-TIFA was detected by Western blotting using rabbit anti-TIFA or anti-HA polyclonal antibody, respectively. BSR-T7/5 cells were infected with rSS1GFP or rSS1GFP-M/NLSm at an MOI of 1 at 36 hpt, and the virus titers were examined at 12 and 24 hpi, respectively.

### siRNA treatment and virus infection

Three pairs of siRNA (Supplemental material Table S8) were designed to knockdown TIFA in BSR-T7/5 cells. For transfection with the siRNA against TIFA, BSR-T7/5 cells were transfected with 25 pmol TIFA siRNA using 7.5 μL Lipofectamine® RNAiMAX (ThermoFisher Scientific, USA) in Opti-MEM medium. The knockdown efficiency was measured by detecting endogenous protein expression by Western blotting analysis after 48 hpt. To study the effect of TIFA on the replication of NDV, rSS1GFP or rSS1GFP-M/NLSm was used to infect TIFA siRNA- or negative siRNA-treated BSR-T7/5 cells at an MOI of 1. The detection of virus titers was performed at 12 and 24 hpi, respectively.

### Statistical analysis

Differences in the expression level of genes, proteins and virus titers between with rSS1GFP- and rSS1GFP-M/NLSm-infected cells were analyzed using SPSS 12.0 software. The independent-samples *t* test was used for data analysis. All experiments were repeated at least three times and the results were shown as the mean ± standard deviation (SD). A *p*-value of < 0.05 was considered significant. *P*-values are indicated by asterisks (*P < 0.05, **P < 0.01, ***P < 0.001).

## Supplementary Material

Supplemental Material

## Figures and Tables

**Abbreviations:** 

NDV	Newcastle disease virus
HRSV	human respiratory syncytial virus
VSV	vesicular stomatitis virus
MeV	measles virus
IAV	influenza A virus
NLS	nuclear localization signal
NES	nuclear export signal
GFP	green fluorescent protein
TMT	tandem mass tag
LC-MS/MS	liquid chromatography tandem-mass spectrometry
DEP	differentially expressed protein
NNSV	non-segmented negative-sense RNA virus
TIFA	TRAF-interacting protein with FHA domain-containing protein A
TRAF6	Tumor necrosis factor receptor-associated factor 6
hpi	hours post-infection
hpt	hours post-transfection
qRT-PCR	quantitative real-time PCR
CPE	cytopathic effect
TCID_50_	50% tissue culture infective dose
siRNA	small interfering RNA
