# Supplementary material for: TMT-based quantitative proteomics analysis reveals the attenuated replication mechanism of Newcastle disease virus caused by nuclear localization signal mutation in viral matrix protein
Source: Virulence. 2020 Jun 27;11(1):607–35. doi: 10.1080/21505594.2020.1770482 (PMC7549962; doi:10.1080/21505594.2020.1770482)
Supplement: Supplemental Material [file KVIR_A_1770482_SM7698.zip › Supplemental material Table S3-S8.doc]

**Table S3 Significantly DEPs shared by rSS1GFP and rSS1GFP-M/NLSm at 12 hpi**

| Protein accession | Protein description | Gene symbol | rSS1GFP | |  | rSS1GFP-M/NLSm | |
| --- | --- | --- | --- | --- | --- | --- | --- |
|  |  |  | Ratio^a^ | *p*-value^b^ |  | Ratio^a^ | *p*-value^b^ |
| G3HDB5 | Serine/threonine-protein kinase Sgk2 | SGK2 | 1.869 | 0.040174 |  | 1.233 | 0.009452 |
| G3IJM1 | TRAF-interacting protein with FHA domain-containing protein A | TIFA | 1.803 | 0.030791 |  | 1.322 | 0.046455 |
| G3HDU9 | Histone H1.1 | H1_1 | 1.701 | 0.014984 |  | 1.614 | 0.026555 |
| G3I695 | Macoilin | TMEM57 | 1.636 | 0.045843 |  | 1.244 | 0.040611 |
| G3HF56 | Calponin | CPN | 1.525 | 0.010180 |  | 0.754 | 0.035169 |
| G3H493 | UPF0459 protein C19orf50-like | RP-L40e | 1.478 | 0.005448 |  | 0.806 | 0.006828 |
| G3I7P0 | Dipeptidyl-peptidase 1 | CTSC | 1.412 | 0.020191 |  | 0.780 | 0.031430 |
| G3II26 | Syntaxin-5 | TPR | 1.360 | 0.048529 |  | 0.770 | 0.011046 |
| G3H4L4 | Serine/threonine-protein kinase Nek10 | SLC4A8 | 1.318 | 0.026281 |  | 1.247 | 0.016183 |
| G3HLW5 | RAB5C | RAB5C | 1.325 | 0.016818 |  | 1.213 | 0.010288 |
| G3IL75 | Collagen alpha-1(V) chain | COL5A1 | 1.273 | 0.014047 |  | 1.260 | 0.021040 |
| G3HQH7 | Olfactomedin-like protein 3 | OLP3 | 1.270 | 0.010236 |  | 1.226 | 0.009788 |
| G3H1A4 | Sidoreflexin | SRF | 1.269 | 0.022467 |  | 1.236 | 0.037637 |
| G3HAC7 | SEC14 domain and spectrin repeat-containing protein 1 | SSRCP1 | 1.266 | 0.019494 |  | 1.2235 | 0.001132 |
| G3IKL1 | Cleft lip and palate transmembrane protein 1-like protein | CRR9p | 1.250 | 0.030012 |  | 1.222 | 0.010233 |
| G3HW17 | Cell division cycle 5-like protein | CDC5L | 1.243 | 0.000402 |  | 1.239 | 0.040423 |
| G3HPV5 | Histone H1.5 | H1_5 | 1.227 | 0.006031 |  | 1.473 | 0.001086 |
| G3HAQ4 | Ubiquinol-cytochrome c reductase complex chaperone CBP3-like | CBP3 | 1.219 | 0.035275 |  | 1.2185 | 0.030776 |
| G3HN29 | Pre-mRNA-splicing regulator WTAP | WTAP | 1.203 | 0.026133 |  | 1.211 | 0.035935 |
| G3I100 | 60S ribosomal protein L27 | RBL27 | 0.802 | 0.007049 |  | 0.830 | 0.004082 |
| G3HX39 | Phosphoglycerate kinase | PGK | 0.776 | 0.047680 |  | 0.815 | 0.009816 |
| G3HYH0 | Serine/threonine-protein phosphatase 6 regulatory subunit 1 | SAPS1 | 0.761 | 0.006114 |  | 0.802 | 0.006109 |
| G3IEG2 | Serpin B6 | SB6 | 0.759 | 0.005138 |  | 0.649 | 0.002313 |
| G3HNR0 | Flavin reductase | BLVRB | 0.746 | 0.002012 |  | 0.785 | 0.038394 |
| G3I3C7 | Putative allantoicase | ALLC | 0.746 | 0.036193 |  | 0.778 | 0.016077 |
| G3IN18 | 60S ribosomal protein L8 | RPL8 | 0.746 | 0.038874 |  | 0.815 | 0.004297 |
| G3ILF3 | Glutathione S-transferase | GST | 0.735 | 0.004765 |  | 0.701 | 0.013562 |
| G3H349 | Serine/threonine-protein phosphatase 2A | PPP2R5 | 0.725 | 0.025535 |  | 0.812 | 0.035123 |
| G3I184 | Trafficking protein particle complex subunit 2 | TRAPPC2 | 0.721 | 0.023800 |  | 0.738 | 0.033505 |
| G3H3T2 | Mitogen-activated protein kinase kinase kinase 7-interacting protein 2 | MAP3K7IP2 | 0.720 | 0.019444 |  | 0.825 | 0.043056 |
| G3HHT8 | 40S ribosomal protein S5 | RPS5 | 0.711 | 0.008579 |  | 0.823 | 0.009119 |
| G3H5N3 | 60S ribosomal protein L29 | RPL29 | 0.667 | 0.038259 |  | 0.817 | 0.040208 |
| G3I3T1 | Beta-soluble NSF attachment protein | BSNAP | 0.659 | 0.007587 |  | 0.819 | 0.046398 |
| G3INX0 | Histone H2B | H2B | 0.548 | 0.028310 |  | 0.618 | 0.012945 |
| G3ILI7 | 26S proteasome non-ATPase regulatory subunit 3 | PSMD3 | 0.529 | 0.017975 |  | 0.652 | 0.047559 |

^a^ means average value of rSS1GFP- or rSS1GFP-M/MNLSm-infected group relative to the mock control group.

^b^ means one-sample *t* test *p-*value.

**Table S4 Significantly DEPs shared by rSS1GFP and rSS1GFP-M/NLSm at 24 hpi**

^a^ means average value of rSS1GFP- or rSS1GFP-M/MNLSm-infected group relative to the mock control group.

| Protein accession | Protein description | Gene symbol | rSS1GFP | |  | rSS1GFP-M/NLSm | |
| --- | --- | --- | --- | --- | --- | --- | --- |
|  |  |  | Ratio^a^ | *p*-value^b^ |  | Ratio^a^ | *p*-value^b^ |
| G3HZW3 | 60S ribosomal protein L18 | RPL18 | 4.169 | 0.0411419 |  | 1.286 | 0.037325 |
| G3IH79 | 60S ribosomal protein L34 | RPL34 | 3.249 | 0.0140065 |  | 1.334 | 0.001499 |
| G3I196 | 60S ribosomal protein L14 | RPL14 | 2.997 | 0.0186150 |  | 1.208 | 0.047185 |
| G3HSH4 | 60S ribosomal protein L24 | RPL24 | 2.885 | 0.0057202 |  | 1.264 | 0.042343 |
| G3HNS5 | Zinc finger protein 512 | ZNF512 | 2.577 | 0.0359014 |  | 1.374 | 0.014925 |
| G3I071 | 60S ribosomal protein L35 | RPL35 | 2.513 | 0.0166971 |  | 1.315 | 0.035513 |
| G3HYH7 | 60S ribosomal protein L28 | RPL28 | 2.499 | 0.0232093 |  | 1.380 | 0.015585 |
| G3I8X0 | 60S ribosomal protein L19 | RPL19 | 2.344 | 0.0059727 |  | 1.249 | 0.0108130 |
| G3HH35 | 60S ribosomal protein L36a | RPL36A | 2.282 | 0.0076485 |  | 1.261 | 0.030377 |
| G3HFG4 | 60S ribosomal protein L4 | RPL4 | 2.096 | 0.0083015 |  | 1.224 | 0.046569 |
| G3IPY4 | 60S ribosomal protein L18a | RPL18A | 1.920 | 0.0044282 |  | 1.293 | 0.014313 |
| G3H1F4 | Ribosomal protein L15 | RPL15 | 1.917 | 0.0239958 |  | 1.201 | 0.002498 |
| G3H0Q0 | 40S ribosomal protein S6 | RPS6 | 1.897 | 0.0429739 |  | 1.258 | 0.020278 |
| G3H5N3 | 60S ribosomal protein L29 | RPL29 | 1.847 | 0.0305984 |  | 1.418 | 0.011640 |
| G3H8N4 | Lactase-like protein | LCTL | 1.837 | 0.0059079 |  | 1.211 | 0.004585 |
| G3GRR8 | 60S ribosomal protein L13 | RPL13 | 1.841 | 0.011682 |  | 1.241 | 0.032648 |
| G3IBK4 | 60S ribosomal protein L10 | RPL10 | 1.750 | 0.0334381 |  | 1.250 | 0.009204 |
| G3IN18 | 60S ribosomal protein L8 | RPL8 | 1.642 | 0.0179714 |  | 1.242 | 0.021739 |
| G3HV18 | 60S ribosomal protein L12 | RPL12 | 1.606 | 0.0214886 |  | 1.248 | 0.010417 |
| G3IN30 | Serine/threonine-protein kinase PLK | PLK1 | 1.572 | 0.0171756 |  | 1.415 | 0.002120 |
| G3HSL4 | Elongation factor 2 | EF2 | 1.540 | 0.0480291 |  | 1.414 | 0.018748 |
| G3I5N5 | DNA topoisomerase 2 | TOP2 | 1.538 | 0.0123537 |  | 1.342 | 0.004845 |
| G3HKF6 | Brix domain-containing protein 2 | BRX1 | 1.521 | 0.0095363 |  | 1.269 | 0.040977 |
| G3I6W1 | Kinesin-like protein | KIF11 | 1.481 | 0.0085077 |  | 1.232 | 0.032075 |
| G3HEA6 | Targeting protein for Xklp2 | TPX2 | 1.460 | 0.0407674 |  | 1.344 | 0.005582 |
| G3HEZ8 | Kinesin-like protein KIF20A | KIF20A | 1.455 | 0.0017188 |  | 1.387 | 0.040029 |
| G3HCT1 | Importin subunit alpha | KPNA2 | 1.412 | 0.0127479 |  | 1.342 | 0.030936 |
| G3H7B6 | WD repeat and HMG-box DNA-binding protein 1 | WDHD1 | 1.395 | 0.0248978 |  | 1.263 | 0.036634 |
| G3I0R8 | Actin-binding protein anillin | ANLN | 1.393 | 0.0028715 |  | 1.253 | 0.005189 |
| G3I4J2 | Kinesin-like protein | KIF11 | 1.389 | 0.0421318 |  | 1.308 | 0.022171 |
| G3HRN7 | Protein timeless-like | TIMELESS | 1.382 | 0.0144625 |  | 1.201 | 0.030396 |
| G3H3U9 | Kinesin-like protein KIF20B | KIF20 | 1.381 | 0.0101376 |  | 1.258 | 0.019873 |
| G3H9C5 | Lymphocyte-specific helicase | LSH | 1.375 | 0.0290909 |  | 1.316 | 0.003039 |
| G3HLU1 | Ubiquitin-conjugating enzyme E2 C | UBE2C | 1.363 | 0.0454879 |  | 1.560 | 0.043924 |
| G3GU69 | Citron Rho-interacting kinase | CIT | 1.359 | 0.0462104 |  | 1.293 | 0.012606 |
| G3HLL8 | Kinesin-like protein | KIF11 | 1.331 | 0.0030053 |  | 1.216 | 0.019737 |
| G3I7Y3 | LAMP1 | LAMP1 | 1.316 | 0.0216648 |  | 0.830 | 0.044264 |
| G3I2K8 | RRM2 | RRM2 | 1.288 | 0.0275728 |  | 1.389 | 0.043018 |
| G3HEH7 | Semaphorin-7A | SEMA7 | 1.276 | 0.0278322 |  | 1.320 | 0.042671 |
| G3HRM7 | DNA primase | PRI1 | 1.260 | 0.0083366 |  | 1.201 | 0.034569 |
| G3IK71 | INCENP | INCENP | 1.260 | 0.0226280 |  | 1.347 | 0.047951 |
| G3H3R6 | Kinesin-like protein | KIF23 | 1.256 | 0.0181210 |  | 1.217 | 0.030012 |
| G3H7M2 | DNA ligase 1 | LIG1 | 1.235 | 0.0110121 |  | 1.212 | 0.042265 |
| G3HCI2 | E3 ubiquitin-protein ligase UHRF1 | UHRF1 | 1.235 | 0.0032389 |  | 1.430 | 0.037063 |
| G3IL75 | Collagen alpha-1(V) chain | COL5A1 | 1.226 | 0.0097879 |  | 1.254 | 0.032309 |
| G3ICK8 | DnaJ-like subfamily A member 1 | DNAJB1 | 1.210 | 0.0347107 |  | 1.233 | 0.000811 |
| G3HW17 | Cell division cycle 5-like protein | CDC5L | 0.830 | 0.0204819 |  | 0.805 | 0.046613 |
| G3HXT9 | Translocator protein | TSPO | 0.830 | 0.0324910 |  | 0.783 | 0.039591 |
| G3H125 | Neutral amino acid transporter A | SLC1A4 | 0.815 | 0.0227133 |  | 0.819 | 0.014652 |
| G3GWG7 | High affinity cationic amino acid transporter 1 | SLC7A1 | 0.814 | 0.0417690 |  | 0.829 | 0.009650 |
| G3IIX9 | DnaJ-like subfamily B member 14 | DNAJB14 | 0.803 | 0.0180685 |  | 0.783 | 0.040256 |
| G3ILI7 | 26S proteasome non-ATPase regulatory subunit 3 | PSMD3 | 0.790 | 0.0284991 |  | 1.329 | 0.002257 |
| G3IHN6 | 4F2 cell-surface antigen heavy chain | SLC3A2 | 0.779 | 0.0436457 |  | 0.804 | 0.040995 |
| G3HFF0 | Solute carrier family 2, facilitated glucose transporter member 1 | SLC2A1 | 0.760 | 0.0335747 |  | 1.200 | 0.023859 |
| G3I278 | Laminin subunit beta-1 | LAMB1 | 0.753 | 0.0172643 |  | 0.830 | 0.021072 |
| G3H0Q1 | Transmembrane protein 50A | TMEM50A | 0.734 | 0.0265848 |  | 0.766 | 0.037231 |
| G3HTV9 | Acyl-coenzyme A oxidase | AOX | 0.721 | 0.0265742 |  | 0.782 | 0.019834 |
| G3HX39 | Phosphoglycerate kinase | PGKB | 0.705 | 0.0170213 |  | 0.760 | 0.007895 |
| G3HAP6 | Acetyl-coenzyme A synthetase | ACSS | 0.689 | 0.0268700 |  | 0.794 | 0.010076 |
| G3H1D5 | Carboxypeptidase | SCPEP1 | 0.664 | 0.0076112 |  | 0.823 | 0.028846 |
| G3HLR4 | GHITM | GHITM | 0.641 | 0.0148322 |  | 0.773 | 0.009709 |
| G3HLX3 | Alpha-N-acetylglucosaminidase | NAGLU | 0.624 | 0.0296712 |  | 0.780 | 0.024513 |
| G3HU02 | Olfactomedin-like protein 2B | OLFML2B | 0.614 | 0.0061889 |  | 0.791 | 0.010753 |
| G3HMN0 | COL8A1 | COL8A1 | 0.569 | 0.0087873 |  | 0.791 | 0.033523 |
| G3IKN8 | Platelet-derived growth factor receptor alpha | PDGFRα | 0.576 | 0.0089487 |  | 0.752 | 0.035904 |
| G3GU43 | T-box transcription factor TBX3 | TBX3 | 0.564 | 0.0269503 |  | 0.705 | 0.039716 |
| G3I1H5 | Legumain | LGMN | 0.529 | 0.0252791 |  | 0.823 | 0.035893 |
| G3IKA3 | Perilipin | PLIN | 0.527 | 0.0126210 |  | 0.647 | 0.042537 |
| G3IBS4 | Protein-L-isoaspartate O-methyltransferase domain-containing protein 2 | PCMTD1 | 0.456 | 0.0050439 |  | 0.783 | 0.015974 |
| G3IJM1 | TRAF-interacting protein with FHA domain-containing protein A | TIFA | 0.271 | 0.0295203 |  | 1.267 | 0.031014 |

^b^ means one-sample *t* test *p-*value.

**Table S5 Top 10 up-regulated and down-regulated significantly DEPs induced by rSS1GFP at 12 hpi and 24 hpi**

| Time (hpi) | Protein accession | Protein description | Gene symbol | Subcellular localization | Ratio^a^ | *p*-value^b^ |
| --- | --- | --- | --- | --- | --- | --- |
| 12 | G3H4Z8 | Heat shock protein A2 | HSPA2 | Cytoplasm | 3.566 | 0.008063 |
|  | G3H843 | TSC22 domain family protein 1 | TSC22D1 | Extracellular | 2.865 | 0.008989 |
|  | G3I7Y3 | Lysosomal Associated Membrane Protein 1 | LAMP1 | Mitochondria | 2.445 | 0.039673 |
|  | G3H8L2 | Dihydrolipoyl dehydrogenase | DLD | Mitochondria | 2.292 | 0.012653 |
|  | G3HUW8 | 40S ribosomal protein S4 | RPS4 | Cytoplasm | 2.199 | 0.001592 |
|  | G3HZ80 | Putative UPF0587 protein | C1PRF123 | Extracellular | 2.163 | 0.009272 |
|  | G3GUJ2 | Tetraspanin | TSPAN9 | Plasma membrane | 2.144 | 0.049907 |
|  | G3HC17 | Interleukin enhancer-binding factor 2 | ILF2 | Mitochondria | 2.021 | 0.008313 |
|  | G3HLY5 | Histone-lysine N-methyltransferase EZH1 | EZH2 | Nucleus | 2.019 | 0.035686 |
|  | G3H2U6 | Splicing factor 3B subunit 4 | SF3B4 | Nucleus | 2.012 | 0.001989 |
|  | G3IIH3 | Eukaryotic translation initiation factor 4E | EIF4E | Extracellular | 0.712 | 0.035337 |
|  | G3H3I0 | Mitochondrial import inner membrane translocase subunit TIM17 | TIM17 | Plasma membrane | 0.698 | 0.024355 |
|  | G3H4F3 | Nuclear receptor subfamily 2 | NR2F6 | Cytoplasm, Nucleus | 0.672 | 0.028824 |
|  | G3H5N3 | 60S ribosomal protein L29 | RPL29 | Cytoplasm, Nucleus | 0.667 | 0.038259 |
|  | G3HFQ7 | Translation initiation factor eIF-2B | EIF2B2 | Cytoplasm, Nucleus | 0.664 | 0.007681 |
|  | G3HAA8 | Putative E3 ubiquitin-protein ligase HERC4 | HERC4 | Extracellular | 0.600 | 0.023333 |
|  | G3IGQ2 | Heat shock cognate 71 | HSC71 | Cytoplasm | 0.531 | 0.035649 |
|  | G3ILI7 | 26S proteasome non-ATPase regulatory subunit 3 | PSMD3 | Cytoplasm, Nucleus | 0.490 | 0.017975 |
|  | G3H247 | Calcium release-activated calcium channel protein 1 | ORAI1 | Plasma membrane | 0.475 | 0.031579 |
|  | G3HR49 | Transcription initiation factor TFIID subunit 8 | TAF8 | Nucleus | 0.425 | 0.0094118 |
| 24 | G3HZW3 | 60S ribosomal protein L18 | RPL18 | Cytoplasm | 4.169 | 0.0411419 |
|  | G3IH79 | 60S ribosomal protein L34 | RPL34 | Cytoplasm | 3.249 | 0.0140065 |
|  | G3I196 | 60S ribosomal protein L14 | RPL14 | Nucleus | 2.997 | 0.0186150 |
|  | G3HSH4 | 60S ribosomal protein L24 | RPL24 | Cytoplasm | 2.885 | 0.0057202 |
|  | G3HNS5 | Zinc finger protein 512 | ZNF512 | Nucleus | 2.577 | 0.0359014 |
|  | G3I071 | 60S ribosomal protein L35 | RPL35 | Nucleus | 2.513 | 0.0166971 |
|  | G3HYH7 | 60S ribosomal protein L28 | RPL28 | Cytoplasm | 2.499 | 0.0232093 |
|  | G3I8X0 | 60S ribosomal protein L19 | RPL19 | Mitochondria | 2.344 | 0.0059727 |
|  | G3HH35 | 60S ribosomal protein L36a | RPL36A | Cytoplasm | 2.282 | 0.0076485 |
|  | G3HFG4 | 60S ribosomal protein L4 | RPL4 | Cytoplasm | 2.096 | 0.0083015 |
|  | G3HMN0 | Collagen Alpha-1(VIII) chain | COL8A1 | Mitochondria | 0.569 | 0.0087873 |
|  | G3GU43 | T-box transcription factor TBX3 | TBX3 | Nucleus | 0.564 | 0.0269503 |
|  | G3IN86 | Dipeptidyl peptidase 7 | DPP7 | Endoplasmic reticulum | 0.560 | 0.0044683 |
|  | G3I5A0 | Short transient receptor potential channel 3 | TRPC3 | Plasma membrane | 0.559 | 0.0465116 |
|  | G3INC5 | Cathepsin L1 | CTSL1 | Extracellular | 0.540 | 0.0481481 |
|  | G3I1H5 | Legumain | LGMN | Extracellular | 0.529 | 0.0252791 |
|  | G3IKA3 | Perilipin | PLIN | Cytoplasm | 0.527 | 0.0126210 |
|  | G3IHQ6 | Sulfurtransferase | MPST | Mitochondria | 0.468 | 0.0352941 |
|  | G3IBS4 | Protein-L-isoaspartate O-methyltransferase domain-containing protein 2 | PCMTD1 | Nucleus | 0.456 | 0.0050439 |
|  | G3IJM1 | TRAF-interacting protein with FHA domain-containing protein A | TIFA | Cytoplasm, Nucleus | 0.271 | 0.0295203 |

^a^ means average value of rSS1GFP-infected group relative to the control group.

^b^ means one-sample *t* test *p-*value.

**Table S6 Top 10 up-regulated and down-regulated significantly DEPs induced by rSS1GFP-M/NLSm at 12 hpi and 24 hpi**

| Time (hpi) | Protein accession | Protein description | Gene symbol | Subcellular localization | Ratio^a^ | *p*-value^b^ |
| --- | --- | --- | --- | --- | --- | --- |
| 12 | G3HDU9 | Histone H1.1 | H1_1 | Nucleus | 1.614 | 0.036555 |
|  | G3HPV5 | Histone H1.5 | H1_5 | Nucleus | 1.473 | 0.010862 |
|  | G3H2E1 | H/ACA ribonucleoprotein complex subunit 2 | NOLA2 | Cytoplasm | 1.354 | 0.031019 |
|  | G3HDW6 | Succinate-semialdehyde dehydrogenase | SSADH | Cytoplasm | 1.322 | 0.008321 |
|  | G3GS61 | PRA1 family protein 3 | PRAF3 | Plasma membrane | 1.296 | 0.047901 |
|  | G3IJ14 | E3 ubiquitin-protein ligase synoviolin | SYVN1 | Plasma membrane | 1.288 | 0.026796 |
|  | G3I8X2 | Long-chain-fatty-acid-CoA ligase 1 | ACSL1 | Cytoplasm | 1.285 | 0.004282 |
|  | G3GXA5 | JAGN1 | JAGN1 | Plasma membrane | 1.269 | 0.037037 |
|  | G3IJM1 | TRAF-interacting protein with FHA domain-containing protein A | TIFA | Cytoplasm, Nucleus | 1.322 | 0.046455 |
|  | G3IL75 | Collagen alpha-1(V) chain | COL5A1 | Cytoplasm | 1.260 | 0.021040 |
|  | G3HF56 | Calponin | CALP | Cytoplasm | 0.754 | 0.035169 |
|  | G3HGY8 | Elongation factor 1-alpha | EEF1A | Cytoplasm | 0.739 | 0.034529 |
|  | G3GYC6 | Profilin-1 | PFN1 | Cytoplasm | 0.705 | 0.012766 |
|  | G3ILF3 | Glutathione S-transferase | GST | Cytoplasm | 0.701 | 0.013562 |
|  | G3HJ80 | Septin-4 | SEPT4 | Cytoplasm | 0.666 | 0.021788 |
|  | G3ILI7 | 26S proteasome non-ATPase regulatory subunit 3 | PSMD3 | Cytoplasm, nucleus | 0.652 | 0.037559 |
|  | G3IEG2 | Serpin B6 | SERPINB6 | Cytoplasm | 0.649 | 0.002313 |
|  | G3INX0 | Histone H2B | H2B | Nucleus | 0.618 | 0.012945 |
|  | G3IEE4 | Protein kinase C delta-binding protein | HSRBC | Extracellular | 0.612 | 0.044783 |
|  | G3HSZ2 | GEMIN6 | GEMIN6 | Cytoplasm, Nucleus | 0.569 | 0.049209 |
| 24 | G3HLU1 | Ubiquitin-conjugating enzyme E2 C | UBE2C | Cytoplasm | 1.560 | 0.043924 |
|  | G3HCI2 | E3 ubiquitin-protein ligase UHRF1 | UHRF1 | Nucleus | 1.430 | 0.037063 |
|  | G3H5N3 | 60S ribosomal protein L29 | RPL29 | Cytoplasm, Nucleus | 1.418 | 0.011640 |
|  | G3IN30 | Serine/threonine-protein kinase PLK1 | PLK1 | Nucleus | 1.415 | 0.002120 |
|  | G3IIG7 | Centromere-associated protein E | CENPE | Nucleus | 1.414 | 0.018748 |
|  | G3HEZ8 | Kinesin-like protein KIF20A | KIF20A | Cytoplasm | 1.387 | 0.040029 |
|  | G3HYH7 | 60S ribosomal protein L28 | RPL28 | Cytoplasm | 1.380 | 0.015585 |
|  | G3HNS5 | Zinc finger protein 512 | ZNF512 | Nucleus | 1.374 | 0.014925 |
|  | G3H278 | 60S ribosomal protein L6 | RPL6 | Plasma membrane | 1.365 | 0.054599 |
|  | G3I2K5 | ODC1 | ODC1 | Cytoplasm | 1.345 | 0.008178 |
|  | G3HTV9 | Acyl-coenzyme A oxidase | ACOX1 | Cytoplasm | 0.782 | 0.019834 |
|  | G3H9N6 | Zinc transporter ZIP6 | ZIP6 | Plasma membrane | 0.777 | 0.020592 |
|  | G3GVR4 | Protein FAM107B | FAM107B | Nucleus | 0.776 | 0.014175 |
|  | G3HLR4 | GHITM | GHITM | Plasma membrane | 0.773 | 0.009709 |
|  | G3H0Q1 | Transmembrane protein 50A | TMEM50A | Plasma membrane | 0.766 | 0.037231 |
|  | G3HX39 | Phosphoglycerate kinase | PGK | Cytoplasm | 0.760 | 0.007895 |
|  | G3IKN8 | Platelet-derived growth factor receptor alpha | PDGFRA | Peroxisome | 0.752 | 0.035904 |
|  | G3INX0 | Histone H2B | H2B | Nucleus | 0.723 | 0.041494 |
|  | G3GU43 | T-box transcription factor TBX3 | TBX3 | Nucleus | 0.705 | 0.039716 |
|  | G3IKA3 | Perilipin | PLIN | Cytoplasm | 0.647 | 0.042537 |

^a^ means average value of rSS1GFP-M/NLSm-infected group relative to the control group.

^b^ means one-sample *t* test *p-*value.

**Table S7 Significantly DEPs associated with RNA transport, RNA degradation, RNA polymerase, basal transcription factors, spliceosome, mRNA surveillance in rSS1GFP- and rSS1GFP-M/NLSm-infected cells**

| Virus | KEGG pathways | Gene symbol | Regulated  Type | Time  (hpi) |
| --- | --- | --- | --- | --- |
| rSS1GFP | RNA transport | - | Up | 12 |
|  |  | ENY2, THOC2, NCBP2, NUP153 | Down |  |
|  |  | - | Up | 24 |
|  |  | ENY2, PAIP1 | Down |  |
|  | RNA degradation | EDC3 | Up | 12 |
|  |  | PAN3, LSM6 | Down |  |
|  |  | - | Up | 24 |
|  |  | - | Down |  |
|  | RNA polymerase | - | Up | 12 |
|  |  | POLR1E, RPB1 | Down |  |
|  |  | RBP4 | Up | 24 |
|  |  | POLR1E, RPABC5 | Down |  |
|  | Basal transcription factors | - | Up | 12 |
|  |  | CCNH, TFIIH4, TAF8 | Down |  |
|  |  | - | Up | 24 |
|  |  | TFIIH1 | Down |  |
|  | Spliceosome | - | Up | 12 |
|  |  | SNRPD1, TXNL4A, TRA2, SNRPB2, SF3B4, ZMAT2, SLU7 | Down |  |
|  |  | SMD1, SF3B4 | Up | 24 |
|  |  | PPIL1, HNRNPA3, SNRPG | Down |  |
|  | mRNA surveillance | - | Up | 12 |
|  |  | CPSF6, SWD2 | Down |  |
|  |  | - | Up | 24 |
|  |  | MSI1, SWD2 | Down |  |
| rSS1GFP-M/NLSm | RNA transport | - | Up | 12 |
|  |  | - | Down |  |
|  |  | ENY2 | Up | 24 |
|  |  | - | Down |  |
|  | RNA degradation | - | Up | 12 |
|  |  | - | Down |  |
|  |  | - | Up | 24 |
|  |  | - | Down |  |
|  | RNA polymerase | RBP4 | Up | 12 |
|  |  | - | Down |  |
|  |  | RBP4, POLR1E | Up | 24 |
|  |  | - | Down |  |
|  | Basal transcription factors | - | Up | 12 |
|  |  | - | Down |  |
|  |  | TAF8 | Up | 24 |
|  |  | - | Down |  |
|  | Spliceosome | - | Up | 12 |
|  |  | - | Down |  |
|  |  | - | Up | 24 |
|  |  | - | Down |  |
|  | mRNA surveillance | - | Up | 12 |
|  |  | - | Down |  |
|  |  | WTAP | Up | 24 |
|  |  | - | Down |  |

- no DEPs were found in this pathway.

**Table S8** The information of TIFA siRNA

| Gene | Name | Sequence(5'→3') | |
| --- | --- | --- | --- |
| TIFA | RNAi#1 | Sense | GCAAAGUGGAAUAUUUAAAUC |
|  |  | Anti-sense | UUUAAAUAUUCCACUUUGCAG |
|  | RNAi#2 | Sense | GGUUAUUCAUCCUAUUUCACC |
|  |  | Anti-sense | UGAAAUAGGAUGAAUAACCCC |
|  | RNAi#3 | Sense | GGAAUAUUUAAAUCAAUAAGG |
|  |  | Anti-sense | UUAUUGAUUUAAAUAUUCCAC |
| Control | Negative RNAi | Sense | UUCUCCGAACGUGUCACGUTT |
|  |  | Anti-sense | ACGUGACACGUUCGGAGAATT |
